# Manipulating spin polarization of titanium dioxide for efficient photocatalysis

**DOI:** 10.1038/s41467-020-14333-w

**Published:** 2020-01-21

**Authors:** Lun Pan, Minhua Ai, Chenyu Huang, Li Yin, Xiang Liu, Rongrong Zhang, Songbo Wang, Zheng Jiang, Xiangwen Zhang, Ji-Jun Zou, Wenbo Mi

**Affiliations:** 1 Key Laboratory for Green Chemical Technology of the Ministry of Education, School of Chemical Engineering and Technology, Tianjin University, Tianjin, 300072 China; 2 Collaborative Innovative Center of Chemical Science and Engineering (Tianjin), Tianjin, 300072 China; 3 Tianjin Key Laboratory of Low Dimensional Materials Physics and Preparation Technology, School of Science, Tianjin University, Tianjin, 300072 China; 4 Tianjin Key Laboratory of Brine Chemical Engineering and Resource Eco-utilization, College of Chemical Engineering and Materials Science, Tianjin University of Science & Technology, Tianjin, 300457 China; 5 Shanghai Synchrotron Radiation Facility, Shanghai Institute of Applied Physics, Chinese Academy of Sciences, Shanghai, 201204 China; 6 Shanghai Synchrotron Radiation Facility, Zhangjiang Lab, Shanghai Advanced Research Institute, Chinese Academy of Science, Shanghai, 201210 China

**Keywords:** Catalyst synthesis, Heterogeneous catalysis, Magnetic materials, Photocatalysis

## Abstract

Photocatalysis has been regarded as a promising strategy for hydrogen production and high-value-added chemicals synthesis, in which the activity of photocatalyst depends significantly on their electronic structures, however the effect of electron spin polarization has been rarely considered. Here we report a controllable method to manipulate its electron spin polarization by tuning the concentration of Ti vacancies. The characterizations confirm the emergence of spatial spin polarization among Ti-defected TiO_2_, which promotes the efficiency of charge separation and surface reaction via the parallel alignment of electron spin orientation. Specifically, Ti_0.936_O_2_, possessing intensive spin polarization, performs 20-fold increased photocatalytic hydrogen evolution and 8-fold increased phenol photodegradation rates, compared with stoichiometric TiO_2_. Notably, we further observed the positive effect of external magnetic fields on photocatalytic activity of spin-polarized TiO_2_, attributed to the enhanced electron-spin parallel alignment. This work may create the opportunity for tailoring the spin-dependent electronic structures in metal oxides.

## Abstract (web summary)

Photocatalyst activity depends significantly on the material’s electronic structures. Here, authors manipulate the electron spin polarization of TiO_2_ by tuning the concentration of Ti vacancies and show improved photocatalytic activities.

## Introduction

Photocatalysis has been regarded as one of the best strategies for hydrogen energy production (from water), environmental remediation (degradation) and synthesis of high value-added chemicals, for which titanium dioxide (TiO_2_) serves as the primary photocatalyst owing to its low cost, inertness, nontoxicity, and strong reducing/oxidizing capabilities^1–4^. The generation of abundant photo-induced electron-hole pairs and their rapid transfer/separation are crucial to maximize the photocatalytic efficiency. To achieve this goal, various strategies have been explored, such as doping with impurity atoms, manipulating exposed facet, introducing vacancies and controlling the morphology and crystal phase^1,5–9^. By these means, the electronic structure of TiO_2_ is tuned to either extend the light absorption range or accelerate the charge separation^1,2,7^. However, in many cases, the intrinsic mechanism behind these results is still unclear, and especially the spin degree of electronic freedom is rarely considered.

The intrinsic characteristics of electrons, such as electron spin properties, could dominate the property of photocatalyst, so the electronic configuration with different spin states may greatly affect the photocatalytic behaviors. Actually, recent work has shown that the performance of some catalysts can be improved by modulating the spin states^10–14^. For example, the structural distortion in atomically thin nanosheets of Co_3_S_4_, NiSe_2_, and NiS results in delocalized spin states that provides not only a high electrical conductivity but also a low adsorption energy of reaction intermediates in oxygen evolution reaction (OER)^11,15^. Also, it has been consolidated that the OER kinetics photocatalyzed by Mn-N-C motifs is dependent on the *e*_g_ occupancy of Mn^3+^ in volcano-type shape with a summit at *ca*. 0.95^16^. Notably, the induced spin polarization by chiral molecules or chiral films (on TiO_2_, Fe_3_O_4_, CuO, etc.) can improve the performances of electrocatalytic and photoelectrochemical (PEC) water splitting^17–20^, by means of suppressing the formation of hydrogen peroxide (H_2_O_2_) and favoring the high yield of paramagnetic triplet molecule oxygen (via the parallel spin alignment of oxygen atoms) due to the chiral-induced spin selectivity (CISS) effect^21–23^. Very recently, the external magnetic field has been applied to strengthen the spin-restricted water oxidation process by accelerating the parallel alignment of oxygen radicals during the formation of O–O bond^24^. Generally, both electrocatalysis and photocatalysis require rapid charge transfer and long lifetime of intermediated species for redox reactions, so the electron spin property is expected to be an intrinsic factor affecting the performance of photocatalyst.

TiO_2_ commonly is a nonmagnetic semiconductor due to the lack of unpaired electrons. However, recent researches show that semiconductors like TiO_2_ and ZnO with abundant metal vacancies exhibit obvious room-temperature ferromagnetism^25–27^, suggesting the appearance of asymmetric spin-up and spin-down channels in these metal-defected oxides. Also, these photocatalysts show considerably improved activity, but the intrinsic mechanism is still unclear. Fortunately, the above results hint a possible way to modulate the electron spin polarization of metal oxides.

Therefore in this work, we regulate the spin polarization of electrons by controlling the content of metal vacancies and clarify the relationship between the spin polarization and photocatalytic performance. The results imply that the best photocatalytic performance of defected Ti_0.936_O_2_ (TiO_2_-10) is closely related to the high degree of spatial spin polarization, *via* enhancing the processes of charge separation and surface reaction. Furthermore, we observe the positive effect of the external magnetic field on the photocatalytic performance of spin-polarized Ti_0.936_O_2_. This work may provide a reliable way for tailoring the spin-dependent electronic structures in metal oxides.

## Results

### Manipulating metal vacancies in TiO_2_

**Fig. 1:**
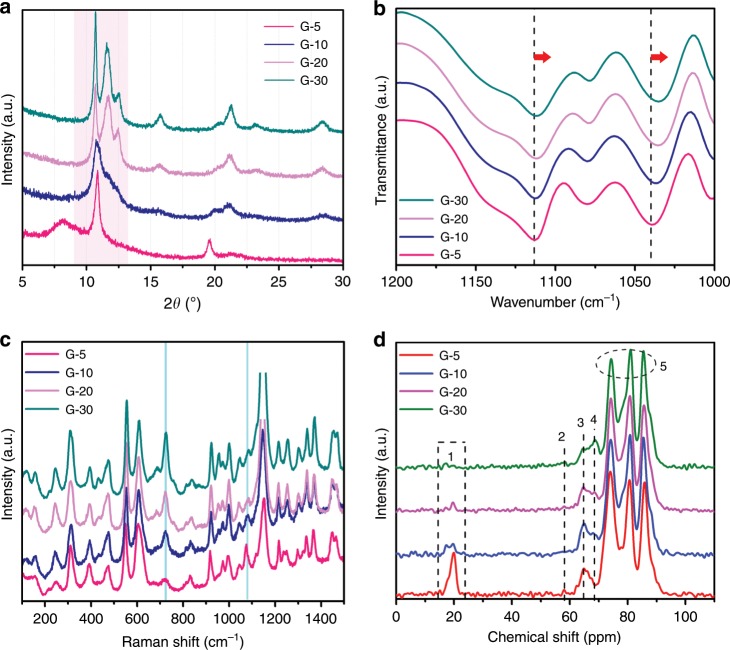
Crystal structures of as-synthesized titanium glycerolates (G-5, G-10, G-20, and G-30). **a** Enlarged XRD patterns (with 2*θ* in the range of 5°–30°), **b** Fourier-transform infrared spectroscopy (FT-IR), **c** Raman spectra, and **d** solid-state CP/MAS ^13^C-NMR spectra.

Previously, we have found that the encapsulation of –CH_2_-OH groups on Ti-O-Ti skeleton can terminate the crystallization along *a*-axis and lead to the formation of lamellar organometallic compounds (LOC, such as metal glycerolate). Importantly, the thermal oxidation of LOC will remove the surface bonding carbon and hydrogen atoms in the form of CO_2_ and H_2_O, leaving the original oxygen atoms linking with skeleton Ti atom and causing the oxygen-rich environment, which finally form the inherent Ti defects during the thermal assembly of Ti-O-Ti parallel lattice chains (Supplementary Fig. 1). Accordingly, we expect that the concentration of metal vacancies might be tuned by adjusting the numbers of glycerol groups in glycerolates (the precursor of defected TiO_2_). Therefore, we synthesized a series of glycerolates (G-*m*) via solvothermal treatment of tetrabutyl titanate (TBT) with glycerol/ethanol through changing the content of glycerol (*m* refers to the volume of glycerol added, mL). During solvothermal synthesis, the glycerol groups replace the butoxy groups of TBT through transesterification reaction. Each glycerol group may share an O atom to connect two adjacent Ti atoms to form parallel chain, and accordingly the chain length and layer spacing will change with the number of glycerol groups. XRD analysis (Fig. 1a and Supplementary Figure 2) shows that all glycerolates have the strongest peak at *ca*. 2*θ* = 10.8° referring to the lamellar peak of metal-based glycerolate^25^. However, the single lamellar peak gradually splits into three peaks with two smaller layer spacings with the increase of *m*. For G-5, the lowest concentration of glycerol groups exists and there are the shortest chain length and largest layer spacing in the unit cell. Then, the chain length becomes longer and the layer spacing becomes smaller with the increase of glycerol dosage from G-5 to G-30, resulting in the better crystallization.

As shown in FT-IR spectra (Fig. 1b and Supplementary Fig. 3), the glycerol C–O stretching vibration bands at 1114 and 1039 cm^−1^ shift to lower wavenumber from G-5 to G-30, indicating that more glycerol groups coordinate to Ti cations^28,29^. And Raman spectra (Fig. 1c and Supplementary Fig. 4) show an obvious growth of peak at 723 cm^−1^ and a decrease of peak at 1077 cm^−1^, also indicating a structure transition in glycerolates. Importantly, the solid-state CP/MAS ^13^C NMR spectra (Fig. 1d) show three well resolved peaks between 70 and 90 ppm (labeled 5) corresponding to the three carbons in glycerolates, in accordance with previous reports^28,29^. Notably, for G-5 there are two small peaks at 20 and 65 ppm (labeled 1 and 3) corresponding to the (–CH_3_, –CH_2_–) and –O-CH_2_– in butoxy groups^30^, in form of terminal groups due to the insufficient transesterification. With the increase of *m*, the peak at 69 ppm (labeled 4) arises gradually, ascribed to more –CH_2_-O– groups in glycerol connecting to the edge Ti atom of glycerolates. The small peak at 58 ppm (labeled 2) belongs to the –CH_2_-OH groups, which is only observed for G-5. The specific locations of carbon peaks in ^13^C NMR spectrum (labeled 1–5) are marked in the molecular structure in Supplementary Fig. 5. The above characterizations confirm the gradually improved crystallization of glycerolates from G-5 to G-30.

**Fig. 2:**
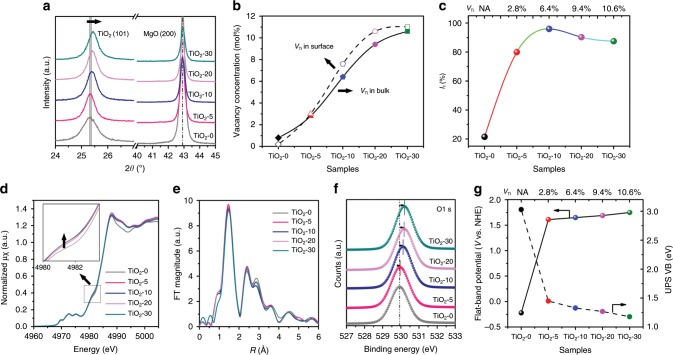
Defect characterizations of metal-defected TiO_2_. **a** Enlarged XRD patterns (with 2*θ* in the range of 24°–45°); **b** Vacancy concentration of TiO_2_ in bulk and surface (determined by chemical titration and XPS analysis, respectively); **c** Intensity of monovacancies from positron annihilation lifetime spectra (PALS), in which *I*_1_ is the relative intensity of *τ*_1_ (metal monovacancies); **d** X-ray absorption near edge structure (XANES) and **e** Fourier transforms of *k*-space oscillations of Ti K edge; **f** high-resolution O1s XPS spectra (fitted); **g** The positions of flat-band potential and UV-photoelectron spectroscopy (UPS) VB (valance band) binding energy.

The glycerolates (G-*m*) were then converted to Ti-defected TiO_2_ (TiO_2_-*m*) upon pyrolysis at 470 °C. Thermogravimetric analysis (Supplementary Fig. 6) shows the weight loss gradually increases from G-5 to G-30, due to the increased amount of glycerol bonding with Ti atoms. XRD patterns (Supplementary Fig. 7) and Raman spectra (Supplementary Fig. 8) confirm that all calcined samples can be identified as anatase TiO_2_ (JCPDS No. 21-1272) without any impurities. What’s more, we used standard periclase to calibrate the XRD patterns, and observed a gradual shift of diffraction peaks at 25.31° to a higher degree (with the *c* axis being gradually narrowed) from TiO_2_-0 to TiO_2_-30 (Fig. 2a, and the lattice parameters in Supplementary Table 1), which is consistent with previous report^25^. SEM (Supplementary Fig. 9) and BET (Supplementary Table 2) characterizations indicate all samples possess similar morphology and comparable surface area. In high-resolution TEM images (Supplementary Fig. 10), the lattice fringes corresponding to (101) facet (*d*_101_ = 0.35 nm) of anatase TiO_2_ is clearly observed^31^.

The Ti/O molar ratios in bulk and surface of TiO_2_ were determined by chemical titration and XPS analysis (the lattice oxygen is fitted from O1s XPS spectra in Supplementary Fig. 11), respectively, and the molar ratio in bulk is very close to that at the surface, indicating the homogenous and uniform composition of defected TiO_2_. As expected, TiO_2_-0 contains almost no Ti vacancies while other samples (*m* = 5–30) possess considerable Ti vacancies, with *V*_Ti_ concentration increasing from 2.8% (TiO_2_-5) to 10.6% (TiO_2_-30) (Fig. 2b and Supplementary Table 1). The defects in TiO_2_ were also determined by positron annihilation lifetime spectra (PALS, Supplementary Table 3). The presence of monovacancies can reduce the surrounding electron density and increase the shortest lifetime component of *τ*_1_^32,33^. All defected TiO_2_ exhibit a longer lifetime *τ*_1_ than TiO_2_-0 (~264 ps), confirming the existence of a large amount of metal monovacancies. What’s more, the relative intensity (*I*_1_) of *τ*_1_ provides the relative concentration of metal monovacancies^34^. It can be seen that the concentration of monovacancies increases from TiO_2_-5 to TiO_2_-10, then decreases for TiO_2_-20 and TiO_2_-30 (Fig. 2c). Correspondingly the intensity of longer lifetime component (*I*_2_ and *I*_3_) referring to positrons captured by larger size defects (such as dual-, tri-defects) or even large voids^32,33^ are increased for the latter two samples. This result means that TiO_2_-10 has the highest concentration of monovacancies, while some vacancies are in form of vacancy clusters at a high *V*_Ti_ concentration (>9%, TiO_2_-20, and TiO_2_-30).

Furthermore, XANES spectra at the Ti K-edge were recorded at room temperature. As shown in Fig. 2d, the defected TiO_2_-*m* samples exhibit similar characteristic lineshape with stoichiometric TiO_2_-0 (anatase), in both regions of pre-edge peaks (4964–4980 eV) and white line peaks (4983–5003 eV), indicating a high phase purity^35^. However, a gradual intensity increase of the pre-edge peak at 4981 eV from TiO_2_-0 to TiO_2_-30 can be observed. Since this peak represents the transition of core electron toward O2p states hybridizing with the empty Ti4p states^35^, the electron numbers of O2p-Ti4p hybrid orbitals should be decreased gradually from TiO_2_-0 to TiO_2_-30, which indicates the presence of lattice O atoms with unsaturated coordination. Without other impurities, this kind of O atoms should be caused by the presence of nearby Ti vacancies. Therefore, the intensity of pre-edge peak (at 4981 eV) is correlated with the Ti vacancies, which rises gradually from TiO_2_-0 to TiO_2_-30.

Meanwhile, the interatomic distances are compared through the Fourier transformed Ti K-edge EXAFS data (Fig. 2e), and all the fitting parameters used by three paths of Ti K-edge EXAFS curves are shown in Supplementary Table 4. A gradual decrease of coordination numbers bonding Ti atoms for Ti_1_ is observed from TiO_2_-0 to TiO_2_-30 as reflected by the amplitude of *R* space (Supplementary Table 4), which verifies the appearance of more Ti vacancies. However, the mean-square disorder of Ti-Ti_1_ distance increases first and then decrease, and TiO_2_-10 owns the highest mean-square disorder, owing to the uniform distribution of monovacancies. The monovacancies will transfer into vacancy clusters in a high defect concentration (such as TiO_2_-30), as confirmed by the decreased mean-square disorder of Ti-Ti distance and PALS results.

Additionally, XPS spectra show that the Ti2p peak is symmetrical (Supplementary Fig. 12), without either shoulder peak appearing at 457.9 eV associated with Ti^3+^ defects or peak at 457.3 eV from Ti–H bonds^7,8^. Moreover, the high-resolution O1s spectra (Fig. 2f) reveal the gradual increase of binding energy from TiO_2_-5 to TiO_2_-30 because the neighbor O atoms of Ti vacancies get less electrons as compared with those on normal sites. Similarly, the DFT calculation shows that the increase of *V*_Ti_ content leads to slight increase of oxygen valence (Supplementary Fig. 13). Moreover, the high-resolution C1s spectra (Supplementary Fig. 14) exclude any C-doped impurities.

Besides, all TiO_2_ samples exhibit similar optical absorption edge at *ca*. 400 nm, with the band gap of *ca*. 3.1 eV (indicated by UV-vis diffuse reflection spectra, Supplementary Figs. 15, 16). Meanwhile, the valence band (VB) (UPS spectra, Fig. 2g and Supplementary Fig. 17) shows a gradual shift from 1.45 eV of TiO_2_-5 to 1.18 eV of TiO_2_-30 (below the Fermi level), much lower than the case of TiO_2_-0 (3.03 eV). The results indicate the energy gap between the Fermi level and VB energy level gradually narrows with the increase of Ti vacancies, which is a *p*-type characteristic^36^. Similarly, a negative slope for defected TiO_2_ in Mott-Schottky plots (Supplementary Fig. 18) also confirms their *p*-type conductivity, while TiO_2_-0 (Supplementary Fig. 19) possessing a normal *n*-type conductivity^8,26^. The position of flat-band potential (*E*_FB_, approximate to the Fermi level)^37^ was further calculated, which shifts from −0.22 V (TiO_2_-0), to 1.61 V (TiO_2_-5) and finally to 1.75 V (TiO_2_-30) vs. NHE (Fig. 2g), similar to UPS VB results. These results confirm the Ti vacancy is a quadruple shallow acceptor that can push down the Fermi level^38^. Moreover, the carrier density was calculated by means of the Mott-Schottky formula (Supplementary Table 5), and the hole density increases from TiO_2_-5 to TiO_2_-30.

### Metal-vacancy-dependent spin polarization of TiO_2_

**Fig. 3:**
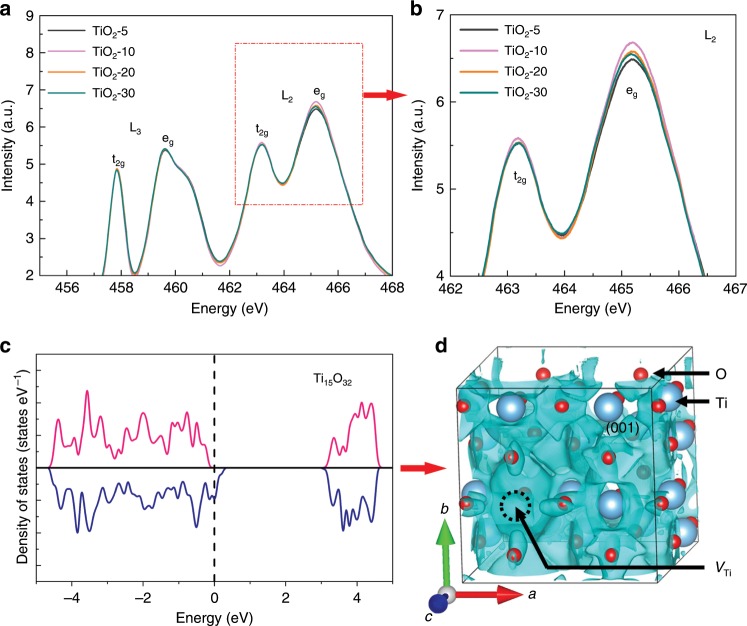
Spin-dependent electronic structures of metal-defected TiO_2_. **a**, **b** Ti L-edge and high-resolution L_2_ XAS spectra. **c** Calculated total density of states (DOS) and **d** (001)-Planar 3D spatial distributions of spin polarization (with the energy interval of [*E*_*F*_-0.2 eV, *E*_*F*_]) of metal-defected Ti_15_O_32_ model (6.25% *V*_Ti_). The specific iso-surface SSP value for **d** is −95%.

The above results show that the concentration of metal vacancies in TiO_2_ can be rationally controlled by adjusting the crystal structure of glycerolate precursors, which provides the possibility to manipulate the electron spin polarization and further the photocatalysis. We applied the Ti L-edge XAS measurements to reveal the complementary electronic properties of Ti compounds, which reflect the transition from Ti2p orbitals into Ti3d and 4s orbitals in the conduction band^39,40^. Importantly, the Ti L edge spectra contain two sets of peaks, L_3_ and L_2_, respectively corresponding to the spin-orbit coupling splitting of initial 2p states into 2p_3/2_ (L_3_) and 2p_1/2_ (L_2_)^39^, which directly refer to the electrons in spin-up (spin quantum number *m*_s_ = +1/2) and spin-down (*m*_s_ = −1/2) states, respectively. As shown in Fig. 3a, b, the TiO_2_-*m* samples show similar L_3_ peak intensity (spin-up electrons), while the L_2_ peak intensity (spin-down electrons) first increases from TiO_2_-5 to TiO_2_-10, and decreases gradually for TiO_2_-20 and TiO_2_-30. The results indicate the increase of Ti vacancies can raise the amount of electrons in spin-down state (or electron polarization), but too much Ti vacancies (like TiO_2_-20 and TiO_2_-30) lead to gradual decrease of electron polarization, may owing to the formation of Ti-vacancies clusters. Notably, the electron polarization of the samples decreases in the order of TiO_2_-10 > TiO_2_-20 > TiO_2_-30 > TiO_2_-5.

In addition, we calculated the spin polarization properties of Ti-defected Ti_15_O_32_, since its defect concentration is similar to TiO_2_-10. As shown in Fig. 3c, the DOS of electron spin-up state is much lower than that of spin-down state around the Fermi level, leading to obvious electron polarization that can provide more spin-down photoinduced electrons under light irradiation. As shown in 3D spatial distributions of spin polarization (Fig. 3d), Ti_15_O_32_ exhibits a large area of negative spatial spin polarization (SSP) in the supercell, which indicates a small possibility of spin polarization reversal in the real space^41^. However, for the defected Ti_35_O_72_ with lower Ti vacancies (2.78%), it shows much lower spin polarization than Ti_15_O_32_ at the Fermi level (Supplementary Fig. 20), which is consistent with the Ti L-edge XAS spectra. The above results verify the existence of metal-vacancy-dependent spin polarization, and the parallel alignment of electron spin polarization may further promote the efficiencies of charge separation and surface reaction for efficient photocatalysis.

### Spin-polarization-dependent photocatalysis

**Fig. 4:**
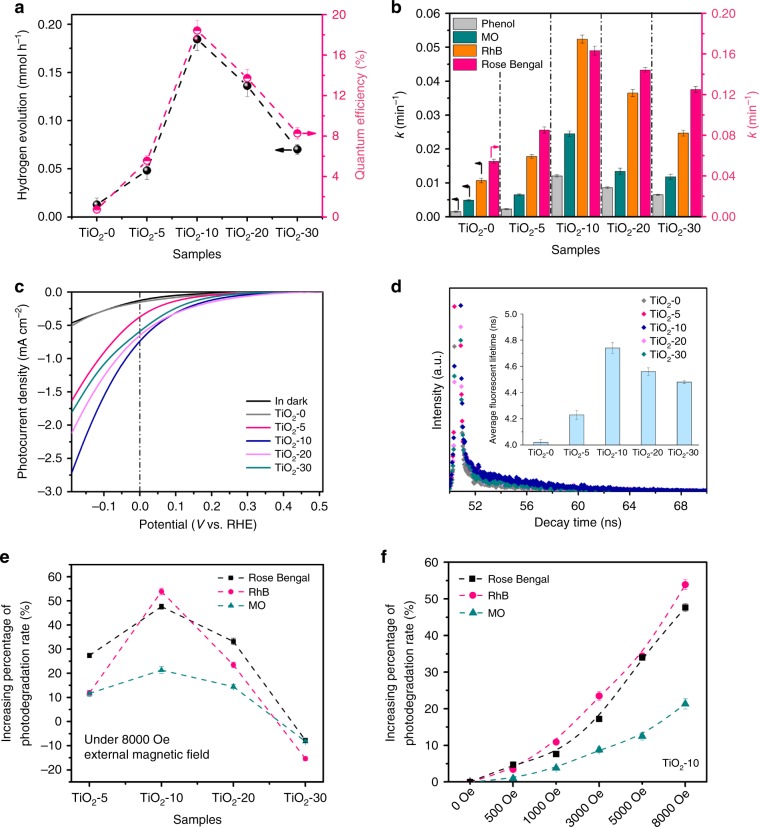
Photocatalytic performance of metal-defected TiO_2_. **a** Photocatalytic H_2_ evolution and quantum efficiency (QE); **b** The degradation rates of four organic pollutants (Phenol, MO, RhB, Rose Bengal); **c** Liner sweep voltammetry (LSV) curves of TiO_2_-*m* (photoelectrodes) under light irradiation and in dark; **d** Time-resolved transient PL decay; **e** Increasing percentage of photodegradation rates of Ti-defected samples under 8000 Oe external magnetic field; **f** Increasing percentage of photodegradation rates of TiO_2_-10 under different strength of magnetic field from 0 to 8000 Oe.

We evaluated the photocatalytic activity of all defected TiO_2_ samples in hydrogen generation and pollutant degradation under simulated solar light irradiation. As shown in Fig. 4a, the defected samples show much higher H_2_ generation rate and quantum efficiency (QE) than normal TiO_2_-0. Notably, the order of activity is TiO_2_-10 > TiO_2_-20 > TiO_2_-30 > TiO_2_-5 > TiO_2_-0, and especially TiO_2_-10 exhibits a 20-fold higher H_2_ generation rate and 18-fold higher QE than TiO_2_-0. Moreover, TiO_2_-10 exhibits high stability without obvious loss in photoactivity after circling experiments (Supplementary Fig. 21). Further for the photodegradation of several organic pollutants, the first-order rate constants (*k*) of all samples are determined and shown in Fig. 4b and Supplementary Figs. 22–25. As expected, the photodegradation rates also show the same activity order as that of the hydrogen generation. Importantly, TiO_2_-10 owns the highest photoactivity, whose photoreaction rate (degradation of phenol) is 8-fold higher than TiO_2_-0.

Besides, TiO_2_-*m* (with *m* = 5–30) with p-type conductivity show high cathodic photocurrent in photoelectrochemical (PEC) water splitting and the activity trend is the same with photocatalysis (Fig. 4c), while n-type TiO_2_-0 can be only applied as photoanode. For n-type TiO_2_-0, the Fermi level (*E*_F_) is higher than electrolyte redox level, leading to electrons transfer from TiO_2_ to electrolyte with upward band bending, while the *E*_F_ of p-type TiO_2_ (like TiO_2_-10) is lower than electrolyte redox level, resulting in electrons transfer from electrolyte to TiO_2_ with downward band bending^42^. Accordingly, n-type TiO_2_-0, with upward band bending at the interface and abundant free electrons, works as the photoanode, while p-type TiO_2_ (like TiO_2_-10), with downward band bending and abundant free holes, can work as photocathode. Moreover, in electrochemical impedance spectroscopy (EIS) under light irradiation (Supplementary Fig. 26), the radius gradually becomes smaller with the increase of Ti vacancies, suggesting that more Ti vacancies (with the majority carrier of holes) will enhance the electrical conductivity, which agrees with the Mott-Schottky results (Supplementary Table 5).

As described above, the photocatalytic activity of defected TiO_2_ is in the order of TiO_2_-10 > TiO_2_-20 > TiO_2_-30 > TiO_2_-5 > TiO_2_-0, which is the same as that of their electron spin polarizations (Fig. 3). Specifically, with the highest spatial spin polarization, TiO_2_-10 shows the best photoactivity. The reason for the positive effect of spin polarization on photocatalysis should be dependent on two aspects: the charges separation and surface reaction.

First, the highly spin-polarized electrons can reduce the recombination of photoinduced electrons and holes during the charge transfer process. When a spin-down electron is excited (to CB), the remaining hole (in VB) also exhibits the same spin-down characteristic and keeps this spin direction unchanged. During the electron transfer, the original spin direction of electrons will lose (and change to spin-up state) due to spin-orbital coupling, hyperfine interaction, etc^43^. Consequently, the recombination will be inhibited because of the lack of spin-up holes under the environment of high spatial spin polarization, which is similar to the giant magnetoresistance effect^44,45^. Therefore, TiO_2_ at *V*_Ti_ = 6.4%, with a highest spin polarization (Fig. 3), can inhibit the recombination of photoinduced carriers to the greatest extent. However, for the stoichiometric TiO_2_ without spin polarization, the excited electron and holes have random spin orientations, and the possibility for one excited electron to recombine with one hole with the same spin orientation is high. As confirmed in the time-resolved transient photoluminescence decay (Fig. 4d and Supplementary Table 6), not surprisingly, TiO_2_-10 possesses the longest fluorescent lifetime, while other samples show relatively short fluorescent lifetime. Especially, the order of fluorescent lifetime changes in the same order of spin polarization, i.e. TiO_2_-10 > TiO_2_-20 > TiO_2_-30 > TiO_2_-5.

Meanwhile for the surface reaction, the spin-polarized electrons play an important role in inhibiting the recombination of active species (free radical) such as hydroxyl radicals. Oxygen-containing species like hydroxyl radicals (·OH) are important active species for photocatalysis, which are formed by donating an electron to the hole of TiO_2_. As discussed above, the spin direction of holes is correlated to the spatial spin polarization, i.e., higher spatial spin polarization leads the higher alignment of hole spin polarization. Therefore, for TiO_2_-10 with wide spatial spin polarization, the spin-polarized holes (note that the SSP at surface is the same as that in the bulk, Supplementary Fig. 27) can only accept the electrons of OH^−^ (from aqueous solution) with the same spin polarization direction (e.g., spin down), leaving the parallel spin alignment among the ·OH species (e.g., spin up). When the single electrons in different ·OH species are in different spin directions, the hydroxyl radical will combine easily to generate H_2_O_2_^17,22^, which will reduce the effective proportion of ·OHs to take part in the surface oxidation reaction. On the contrary, ·OHs with single electrons in the same spin direction are not easy to combine^17,23,46^, thus promoting the surface reactions of water splitting and degradation (especially for the case of TiO_2_-10).

It is worth noting that, the above spin-restricted merits can be further enhanced by external magnetic field^24^. Therefore, a magnetic field (easily by the electromagnet) was applied to investigate its effect on the photocatalytic activity of defected TiO_2_. From Fig. 4e and Supplementary Fig. 28a–c, it can be seen that the magnetic field does enhance the activity of TiO_2_-5, TiO_2_-10, and TiO_2_-20, which may be attributed to the inhibition of the combination between ·OH species with anti-parallel spin directions^17^. And the effect of magnetic field is most significant for TiO_2_-10, with the reaction constant increased by 54% (RhB degradation) at a magnetic field of 8000 Oe, which is related to the its best magnetic feature (0.2 μ_B_/unit cell or 3.2 μ_B_/supercell, Supplementary Figs. 29–31, similar to the previous result^47^) as well as the wideset distribution of spin-polarized electrons^24^. Moreover, for TiO_2_-10, the degree of photocatalytic enhancement increases with the strength of magnetic field (Fig. 4f and Supplementary Fig. 28d–f), further confirming the significant positive effect by magnetic field. However, the photocatalytic activity of TiO_2_-30 is suppressed at an external magnetic field, because the spatial spin polarization reversal (caused by the abundant defects clusters) may appear on the surface, which facilitates the combination of ·OH species.

## Discussion

In summary, we regulated the spin polarization in TiO_2_ by modulating the content of metal vacancies and illustrated that the increase of spatial spin polarization shows positive effect on the photocatalysis. Especially, the introduced *ca*. 6.4% Ti vacancies (TiO_2_-10) leads to intensive spatial spin polarization with wide distribution, which promotes the efficiencies of charge separation and surface reaction via the parallel alignment of electron spin polarizations, and results in significantly promoted photocatalytic activities. Meanwhile, the positive effect of external magnetic fields on Ti-defected TiO_2_ is also observed, attributed to the enhanced electron-spin parallel alignment. This reliable spin-polarization-modulation strategy via defects tuning gives us inspiration to tailor the spin-dependent electronic structures for highly effective catalysts.

## Methods

### Density functional theory calculations

The density functional theory (DFT) calculations were implemented in Vienna Ab initio Simulation Package^48^. DFT with generalized gradient approximation (GGA) provides accurate lattice constants in anatase TiO_2_, however, the band gap of anatase TiO_2_ is underestimated by GGA^25^. In order to improve the band gap, the effective Hubbard *U* of 7.2 eV is applied for the Ti 3*d* states^49^ (see the influence of *U* values on the spatial spin polarization results in Supplementary Fig. 32). Based on the GGA + *U* method, the calculated band gap of anatase TiO_2_ is 3.3 eV, which is very close to the experimental value of 3.2 eV. The supercell size and atomic numbers of normal and 6.25% Ti-defected TiO_2_ are listed in Supplementary Table 7.

The energy cutoff for plane wave basis set is 400 eV. The convergence criteria for the energy and atomic forces are 10^–5^ eV and 0.01 eV Å^−1^, respectively. The Brillouin Zone is sampled with *Γ*-centered 3 × 3 × 3 *k* point meshes for TiO_2_. The spatial spin polarization (SSP) is defined as:

1 $$P(r,z,\varepsilon ) = \frac{{n_s^ \uparrow (r,z,\varepsilon ) - n_s^ \downarrow (r,z,\varepsilon )}}{{n_s^ \uparrow (r,z,\varepsilon ) + n_s^ \downarrow (r,z,\varepsilon )}}$$

where the $n_s^{ \uparrow ( \downarrow )}(r,z,\varepsilon )$ is the spin-up (down) charge density in the real space with an energy interval of [*ε*, *E*_F_]^41^. In this work, the energy range of [*E*_*F*_-0.2 eV, *E*_*F*_] is used (see the effect of energy ranges on SSP results in Supplementary Fig. 33).

### Synthesis of TiO_2_ with metal vacancies

Tetrabutyl titanate [Ti(OC_4_H_9_)_4_, TBT], H_2_PtCl_6_·6H_2_O, and methyl orange (MO) were purchased from Tianjin Guangfu Fine Chemical Research Institute (China); ethanol, glycerol, and methanol were from Tianjin Yuanli Chemical Co., Ltd; Phenol was from J&K Chemical; RhB and Rose Bengal were from Aladdin Industry Corporation; Milli-Q ultrapure water with a resistivity higher than 18.2 MΩ·cm was used in all experiments. All the reagents were analytical grade and used as received.

2 g TBT was added into a solution of *m* (*m* = 0, 5, 10, 20, 30) mL glycerol and (80-*m*) mL ethanol under a magnetic stirring, and the solution was transferred into a 100 mL Teflon-lined autoclave and heated at 180 °C for 24 h. Then, the produced white powders (glycerolates) were collected, washed with absolute ethanol, dried at 60 °C overnight (labeled as **G-*m***), and finally calcined in air at 470 °C for 1 h with a heating rate of 5 °C min^−1^ (labeled as **TiO**_**2**_**-*m***).

### Structure characterizations

X-ray diffraction (XRD) patterns were recorded using Panalytical X’Pert Pro X-ray diffractometer equipped with Cu K*α* radiation at 40 kV and 140 mA at a scanning rate of 5° min^−1^. Scanning electron microscopy (SEM) images were observed using a field-emission scanning electron microscope (Hitachi, S-4800). Transmission electron microscopy (TEM) analysis was carried out using a Tecnai G^2^ F-20 microscope with a field-emission gun operating at 200 kV. X-ray photoelectron spectrum (XPS) analysis was conducted with a PHI-1600 X-ray photoelectron spectroscope equipped with Al K*α* radiation, and the binding energy was calibrated by the C1s peak (284.8 eV) of contamination carbon. Specific surface area (*S*_BET_) was calculated based on N_2_ adsorption/desorption isotherms recorded on a Micromeritics TriStar 3000 instrument at 77 K, and all the samples were outgassed in a vacuum at 150 °C for 12 h. UV-vis diffuse reflectance spectra (UV-vis DRS) were obtained from a Shimadzu UV-2600 spectrometer equipped with a 60 nm diameter integrating sphere using BaSO_4_ as reference. Time-resolved fluorescence anisotropy decays were recorded on a FL3 system (Horiba Scientific, Edison, NJ) utilizing the time-correlated single photon count. Raman spectra was recorded by a Raman Microscope (DXR Microscope, ThermoFisher, USA), with a 100-mW 532-nm line of Nd:YAG laser as the excitation source. Thermogravitity (TG) analysis was conducted on a TGA Q500 thermogravimeter under an air flow with a rate of 5 °C min^−1^. Mott-Schottky (MS) plots were obtained by a capacitance measurement in a standard three-electrode setup (the electrolyte was 0.2 M Na_2_SO_4_ electrolyte; the reference electrode was Ag/AgCl; and a Pt wire was used as the counter electrode; Autolab potentiostat/galvanostat, Model PGSTAT 302N), where TiO_2_ powders (after being grinded) spin-coating on F-doped tin oxide (FTO) glass were used as the working electrode. Electrochemical impedance spectroscopy (EIS) measurements were carried out with a sinusoidal ac perturbation of 10 mV applied over the frequency range of 0.01–10^5^ Hz.

X-ray absorption fine structure spectroscopy (XAFS) was performed at the 1W2B beamline of Beijing Synchrotron Radiation Facility. The Ti L-edge XAS spectra were collected on the beamline BL01C1 in NSRRC. Positron annihilation lifetime spectra (PALS) were measured with a fast/slow coincidence ORTEC system with a time resolution of ~201 ps (full width at half-maximum). Fourier transform infrared (FT-IR) spectra were recorded on a BioRad FTS 6000 spectrometer. All samples were mixed with KBr and pressed into a thin plate for measurement. Solid-state CP/MAS ^13^C-NMR spectrum were tested using Infinityplus 300, Varian, USA.

The concentration of Ti atom (Ti/O ratio) in the bulk of oxide was analyzed by chemical titration method. 0.1 g of the sample was heated slowly to 110 °C for 2 h to evaporate volatile ingredients. Then it was mixed with 4 g sodium peroxide (Na_2_O_2_) and then heated to 800 °C for 5 min to remove residual organic materials. After that, it was transferred to a 300 mL beaker, with further 50 mL water, 30 mL sulfuric acid (9 mol L^−1^) and 30 mL hydrochloric acid (12 mol L^−1^) added. The mixture was heated until it was dissolved completely. After cooling, the solution was transferred to a 500 mL Erlenmeyer flask, and 40 mL hydrochloric acid (0.6 mol L^−1^) was added. After that, 2 g of metallic aluminum was added to reduce Ti (IV) ions to Ti (III) ions. The next stage was the titration of this solution with standardized ferric ammonium sulfate solution to the sharp color change from violet to red. Ammonium thiocyanate solution (NH_4_SCN, 300 g L^−1^) was used as the indicator of the end-point. The titration was repeated at least 3 times for each sample, with the experimental error less than 0.5%. The Ti/O ratio on the surface was calculated according to the surface concentration of Ti and O detected by XPS analysis.

Photoelectrochemical (PEC) properties were measured using a CHI660E electrochemical workstation in a three-electrode cell with a Pt wire as the counter electrode and an Ag/AgCl reference electrode. Na_2_SO_4_ (0.2 M) was used as electrolyte solution. The working electrode was prepared by the spin-coating TiO_2_ slurry on the FTO glass electrode (1 cm × 1 cm) and heating at 300 °C for 1 h. Potentials vs. Ag/AgCl were converted into potentials *vs*. reversible hydrogen electrode (RHE) according to the Nernst equation (*E*_RHE_ = *E*_Ag/AgCl_ + 0.059 pH + 0.196). The *J*–*V* curves were measured with a scanning rate of 10 mV s^−1^ under the irradiation of a xenon lamp (300 W).

### Photocatalytic tests

Photocatalytic hydrogen production was carried out in a top-irradiation Pyrex reaction vessel connected to closed glass gas system. 10 mg catalyst were dispersed in 100 mL aqueous solution containing methanol (30 _vol_%). 0.5 _wt_% Pt was introduced as cocatalyst by in situ photodeposition. The temperature of reaction solution was maintained at 0.5 °C ± 0.1 °C and the light source is a 300 W high-pressure xenon lamp (100 mW cm^−2^, PLS-SXE 300UV). The produced hydrogen was analyzed using an online gas chromatography (Bruker 450-GC, thermal conductive detector, 5 Å molecular sieve column, and N_2_ as carrier gas). For revealing the catalytic reaction pathway difference between n-type TiO_2_-0 and p-type TiO_2_-10, the gas products, and liquid products of the photocatalysis are further determined by gas chromatography and liquid chromatography, respectively, and the results indicate that the reaction products are same (with gas products of CO_2_ and H_2_, and liquid products of formaldehyde, without formic acid formed since its spontaneous decomposition to CO_2_ and H_2_O^50^) whether n-type or p-type TiO_2_ is used as photocatalyst (see the details in Supplementary Fig. 34).

Photodegradation of organic pollutants was conducted in an opening quartz chamber (150 mL) vertically irradiated by a 300 W high-pressure xenon lamp located on the upper position. The light density was controlled at 100 mW cm^−2^. The irradiation area was *ca*. 50 cm^2^. Reaction conditions: temperature, 25 ± 0.2 °C; *C*_0_(MO) = 120 μmol L^−1^; *C*_0_(Phenol) = 400 μmol L^−1^; *C*_0_(RhB) = 20 μmol L^−1^; *C*_0_(Rose Bengal) = 20 μmol L^−1^ and TiO_2_: 0.2 g L^−1^; no acid or alkaline reagents were added. Reaction was conducted by magnetic stirring under atmosphere after stirring for 20 min in darkness to achieve adsorption equilibrium. Samples were withdrawn, centrifuged, and analyzed using UV-vis spectrometer (UV-2600, Shimadzu Ltd.).

### Photocatalytic degradation under magnetic field

Photodegradation of organic pollutants was conducted in a NMR tube (Diameter: 0.5 cm, Length: 18 cm, Content: 2 mL) vertically irradiated by a 300 W high-pressure xenon lamp (100 mW cm^−2^, PLS-SXE 300UV) located on the upper position, using the vibrating sample magnetometer (LDJ 9600). The irradiation area was *ca*. 8 cm^2^. Reaction conditions: temperature, 25 ± 0.2 °C; *C*_0_(MO) = 120 μmol L^−1^; *C*_0_(RhB) = 20 μmol L^−1^; *C*_0_(Rose Bengal) = 20 μmol L^−1^ and TiO_2_: 2 g L^−1^; no acid or alkaline reagents were added. Samples were withdrawn, centrifuged, and analyzed using UV-vis spectrometer (UV-2600, Shimadzu Ltd.).

## Supplementary information

Supplementary Information

## Data Availability

The source data underlying Figs. 1–4 and Supplementary Figs. 2–4, 6–8, 11–19, 21–26, 28, 29, 31 are provided as a Source Data file.

## Source data

source data
