# Supplementary material for: Manipulating spin polarization of titanium dioxide for efficient photocatalysis
Source: Nat Commun. 2020 Jan 21;11:418. doi: 10.1038/s41467-020-14333-w (PMC6972883; doi:10.1038/s41467-020-14333-w)
Supplement: Supplementary file 1 — Supplementary Information [file 41467_2020_14333_MOESM1_ESM.pdf]

Supplementary Information (SI)

Manipulating Spin Polarization of Titanium Dioxide for Efficient Photocatalysis

Pan et al.

Supplementary Figures

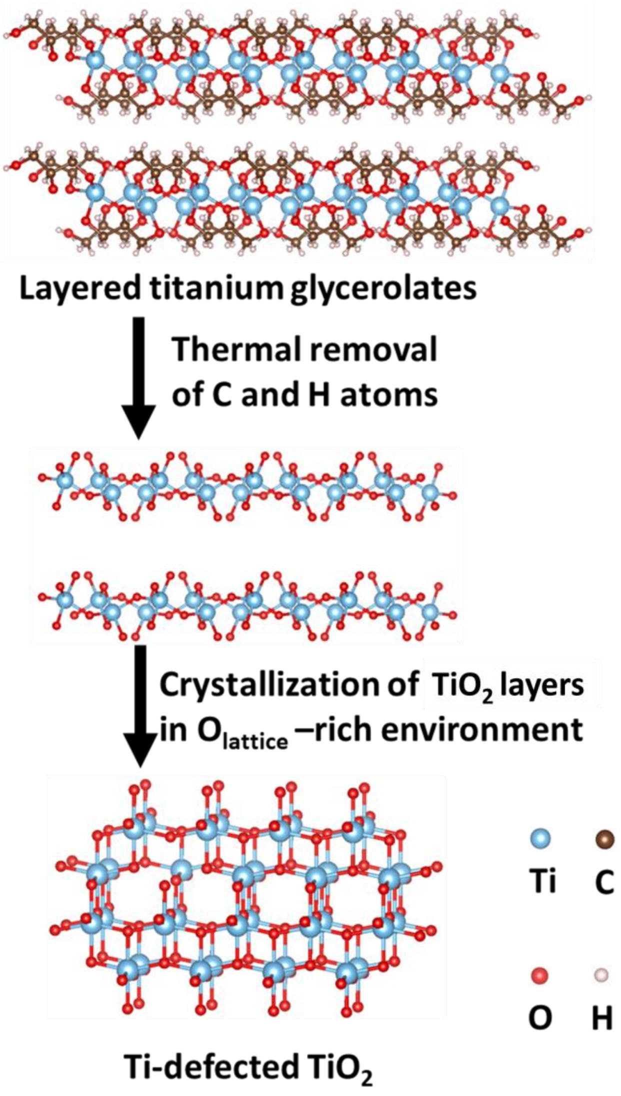

Supplementary Fig. 1 Schematic formation of Ti-defected TiO_2.

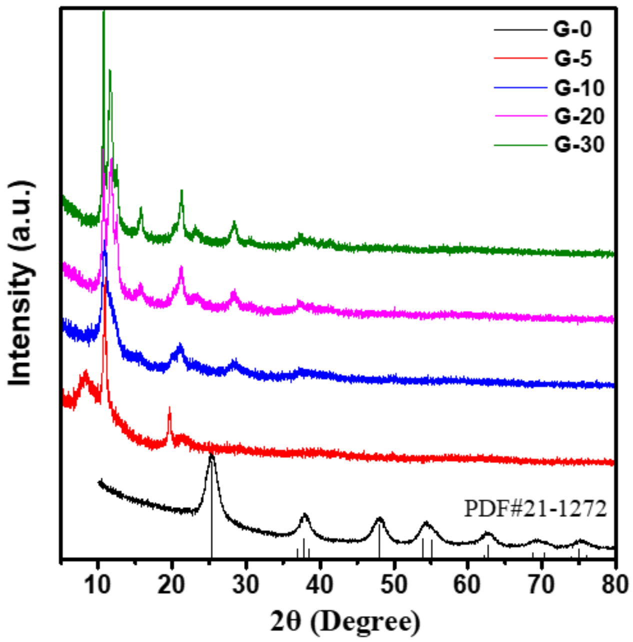

Supplementary Fig. 2 XRD patterns of G-*m*.

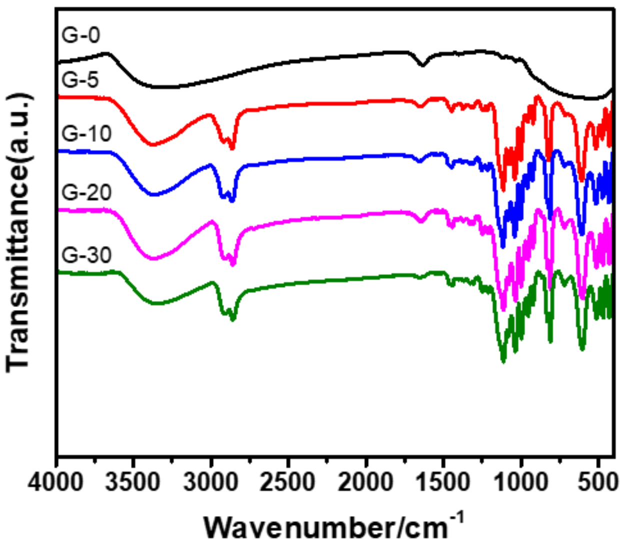

Supplementary Fig. 3 FT-IR spectra of G-*m*.

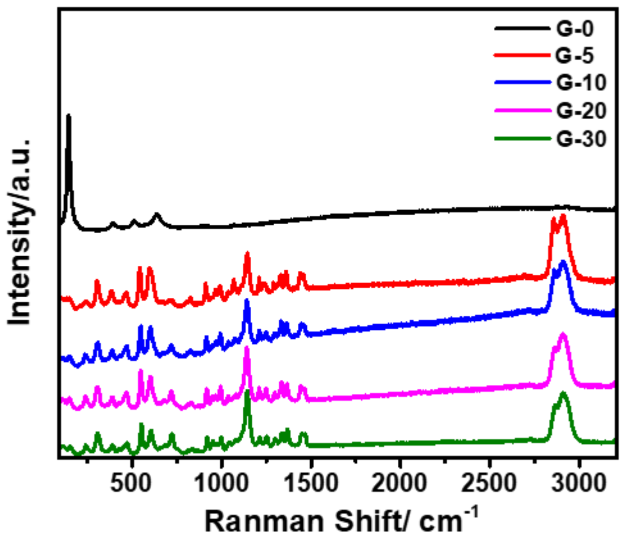

Supplementary Fig. 4 Raman spectra of G-*m*.

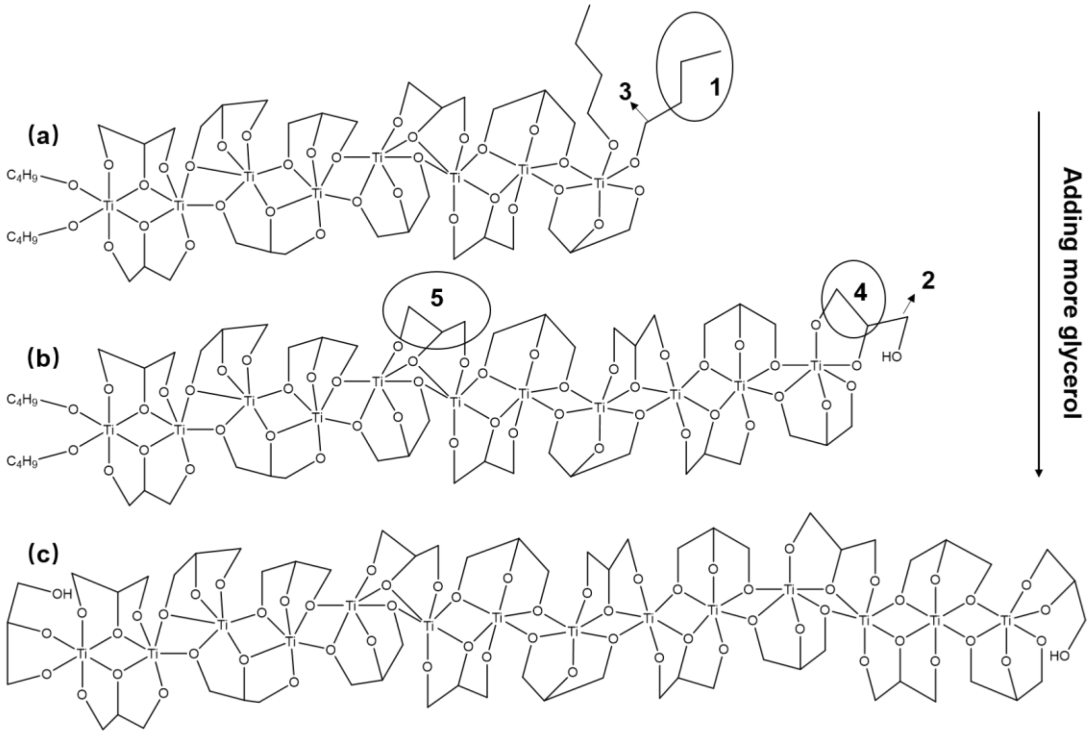

Supplementary Fig. 5 Structure evolution of glycerolates with increasing amount of glycerol.

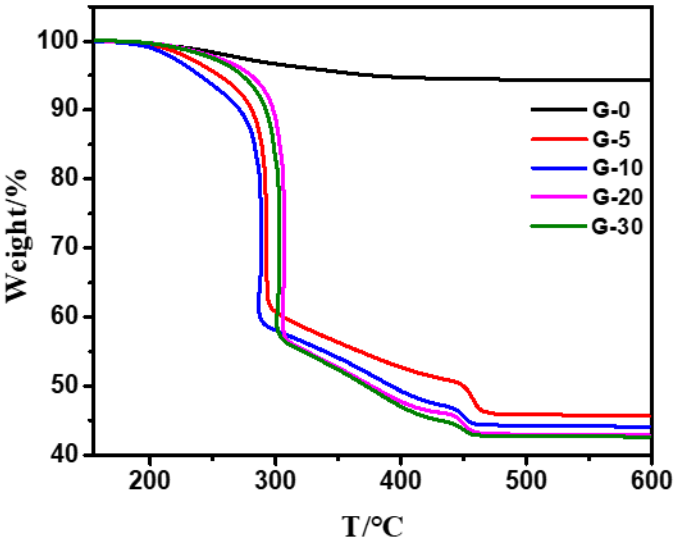

Supplementary Fig. 6 TG curves of G-*m*.

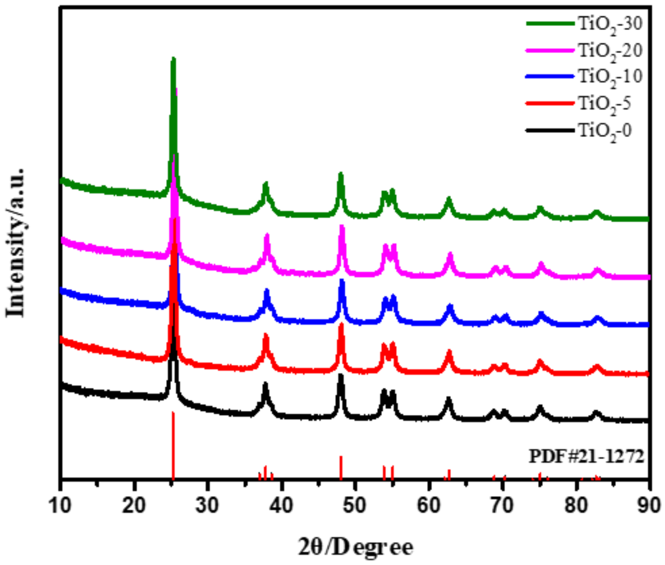

Supplementary Fig. 7 XRD patterns of TiO_2-*m*.

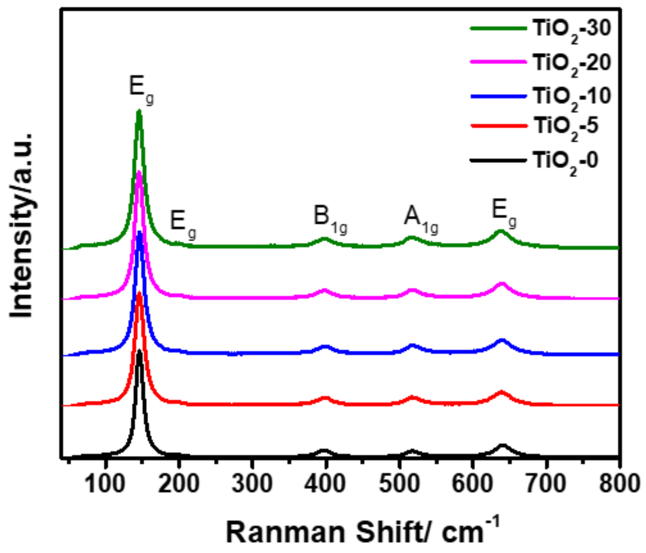

Supplementary Fig. 8 Raman spectra of TiO_2-*m*.

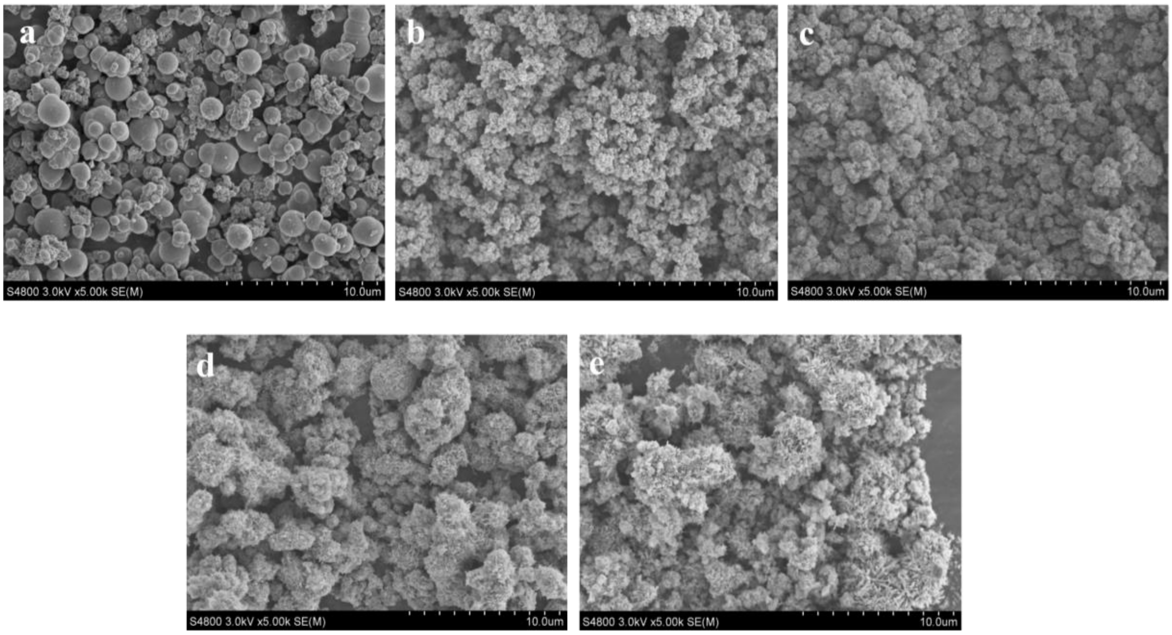

Supplementary Fig. 9 SEM images of TiO_2-*m*. (a) TiO_2-0, (b) TiO_2-5, (c) TiO_2-10, (d) TiO_2-20, and (e) TiO_2-30.

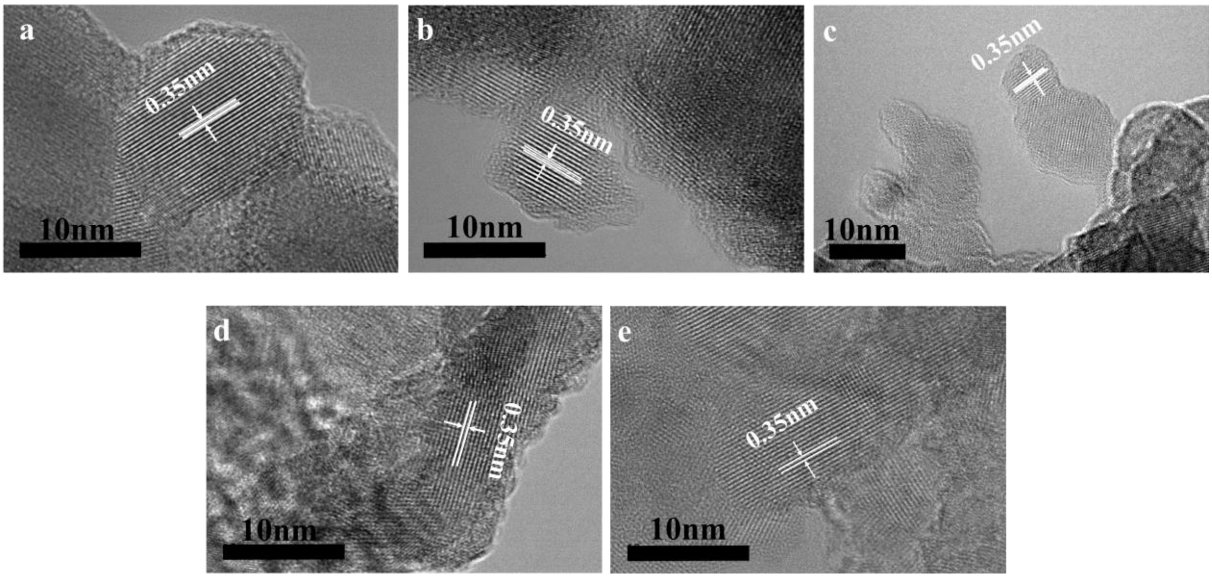

Supplementary Fig. 10 HR-TEM images of TiO_2-*m*. (a) TiO_2-0, (b) TiO_2-5, (c) TiO_2-10, (d) TiO_2-20, and (e) TiO_2-30.

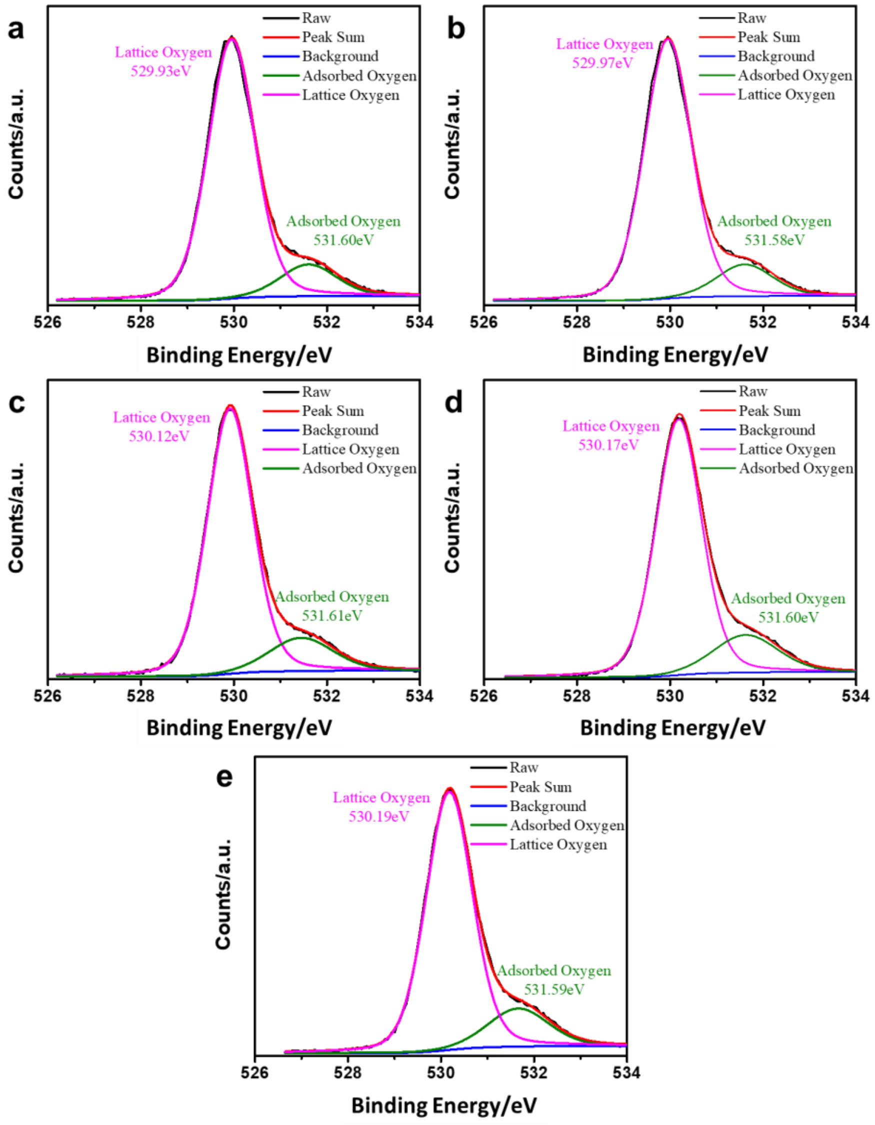

Supplementary Fig. 11 Fitted O1s XPS signals. (a) TiO_2-0, (b) TiO_2-5, (c) TiO_2-10, (d) TiO_2-20, and (e) TiO_2-30, respectively.

The high resolution XPS spectrum of O1s can be divided into two peaks located at *ca.* 531.6 eV and *ca.* 530.1 eV, which are attributed to the adsorbed oxygen and lattice oxygen, respectively [1,2].

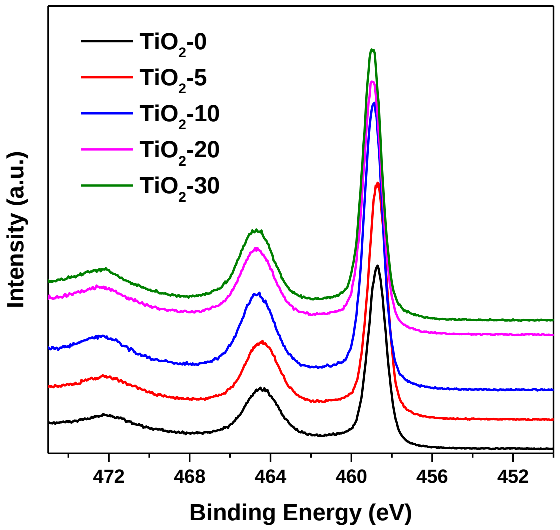

Supplementary Fig. 12 Ti2p XPS spectra of TiO_2-*m*.

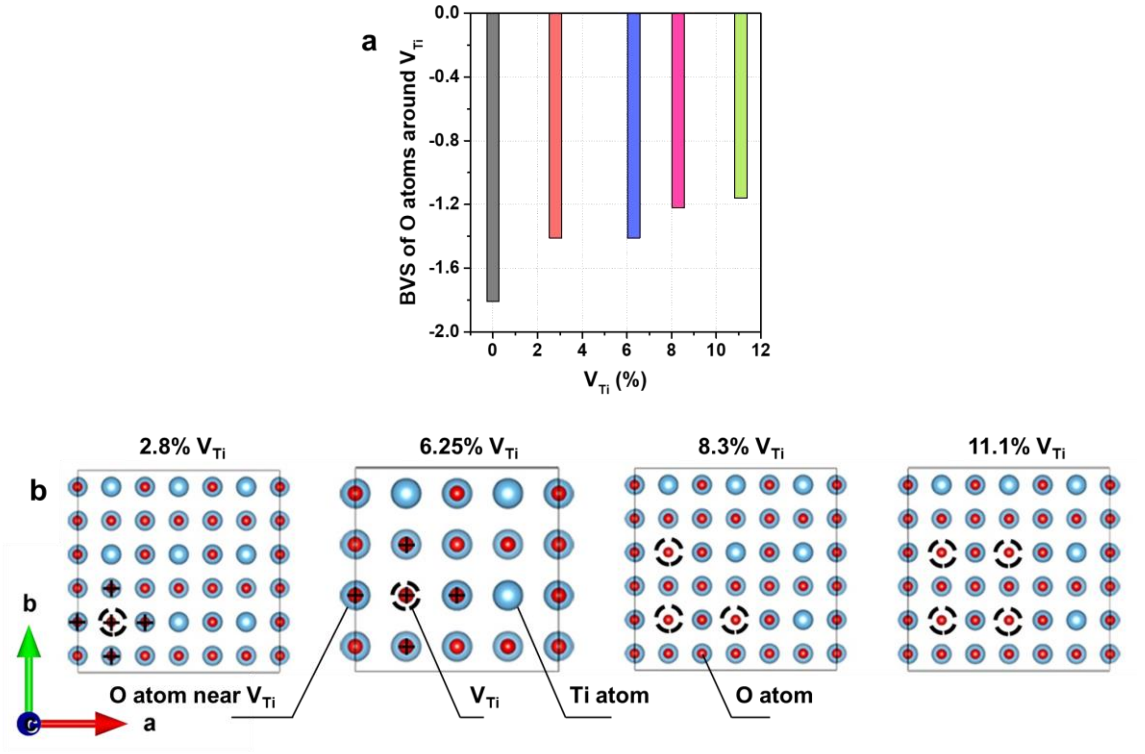

Supplementary Fig. 13 The bond-valence sum calculation. The bond-valence sum (BVS) of O atoms around Ti vacancies in TiO_2 (a), and their geometry structures (b).

BVS is defined as $\mathrm{BVS} = \sum_{i}^{n} \exp\left(\frac{R_0 - R_i}{b}\right)$, with R_0 as the bond-valence parameter and b as 0.37 Å. The R_0 for Ti^{4+} is 1.815, while R_i refers to the ith bond length).

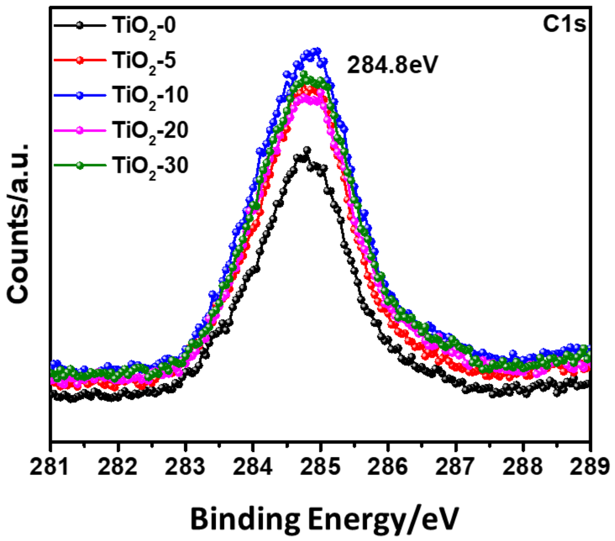

Supplementary Fig. 14 High-resolution C1s XPS spectra of TiO_2-*m*. The observed single peak at 284.8 eV is attributed to adventitious carbon.

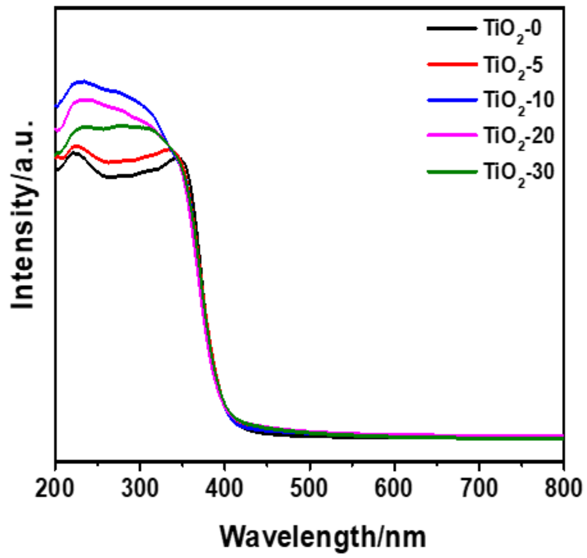

Supplementary Fig. 15 UV-Vis DRS spectra of TiO_2-*m*.

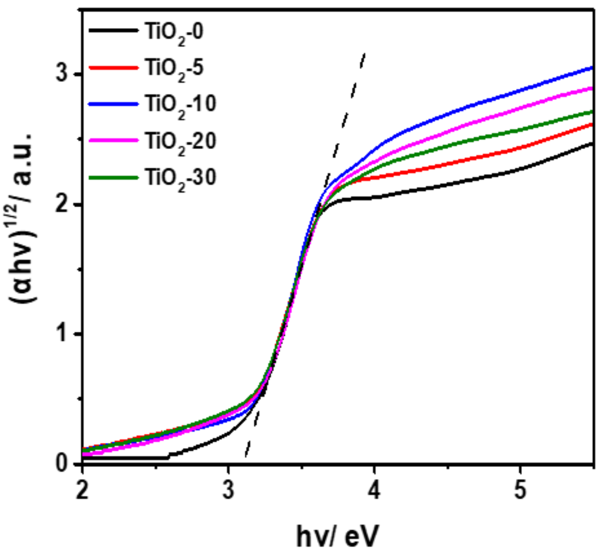

Supplementary Fig. 16 Plots of transformed Kubelka-Munk function *vs.* photo energy of TiO_2-*m*.

The band gap energy was calculated using the following formula:

$$\alpha h\nu = A(h\nu - E_g)^n \quad (1)$$

where α is the absorption coefficient, h is Planck's constant, E_g is the energy band gap, v is the light frequency, and n corresponds to the characteristics of the transition in semiconductors[3].

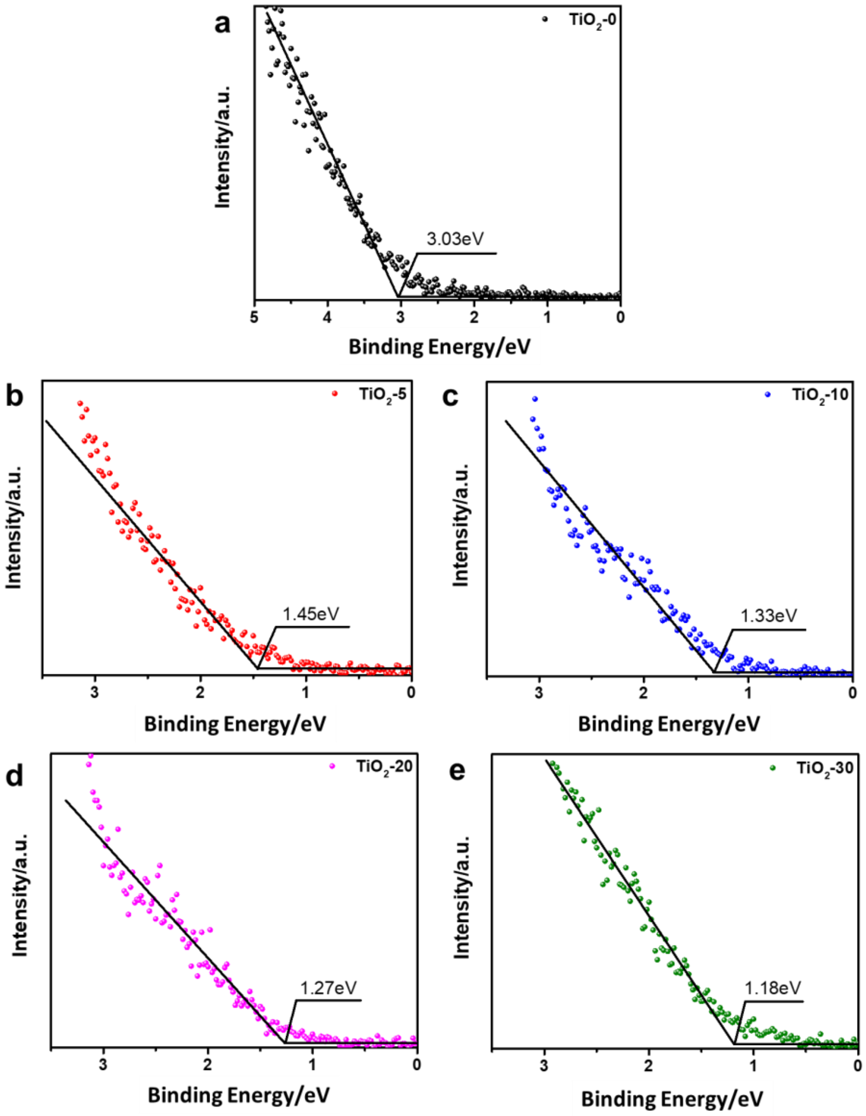

Supplementary Fig 17 UPS valence-band spectra. (a) TiO_2-0, (b) TiO_2-5, (c) TiO_2-10, (d) TiO_2-20, and (e) TiO_2-30.

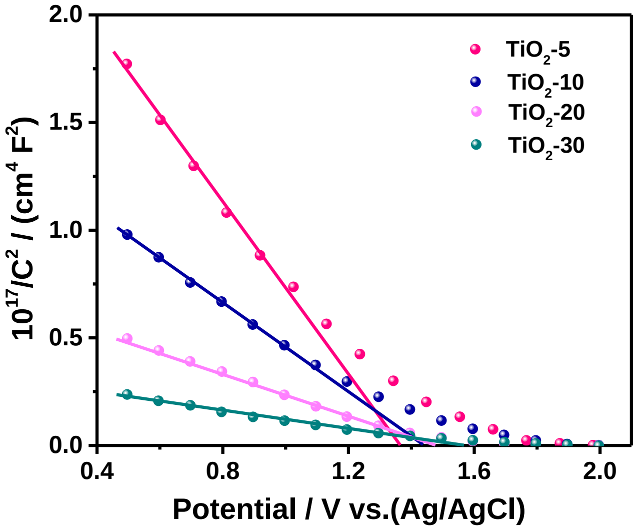

Supplementary Fig. 18 Mott-Schottky plots of TiO_2-*m* measured in a standard three-electrode cell.

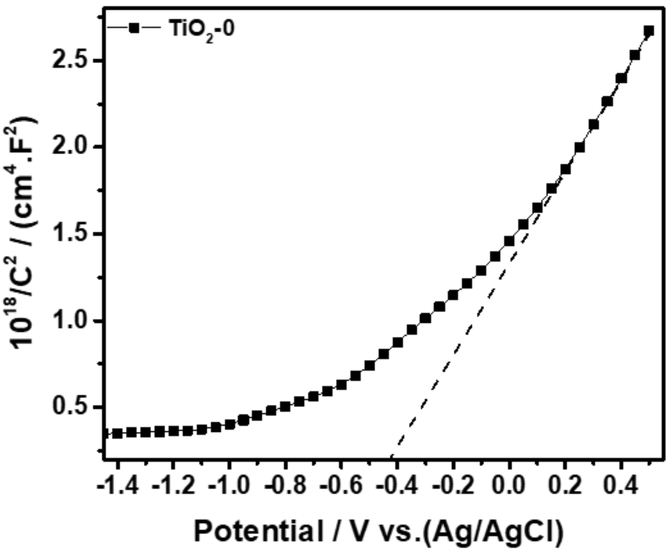

Supplementary Fig. 19 Mott-Schottky plots of TiO_2-0 measured in a standard three-electrode cell.

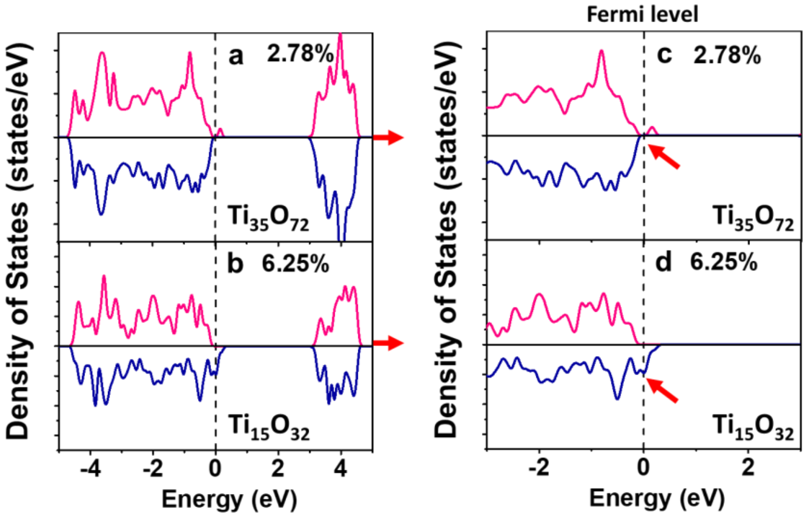

Supplementary Fig. 20 Calculated total density of states. **a**,**c**, $Ti_{35}O_{72}$ (2.78% V_{Ti}) and **b**,**d**, $Ti_{15}O_{32}$ (6.25% V_{Ti}). $Ti_{35}O_{72}$ shows much lower spin polarization than $Ti_{15}O_{32}$ at the Fermi level (marked by the arrays).

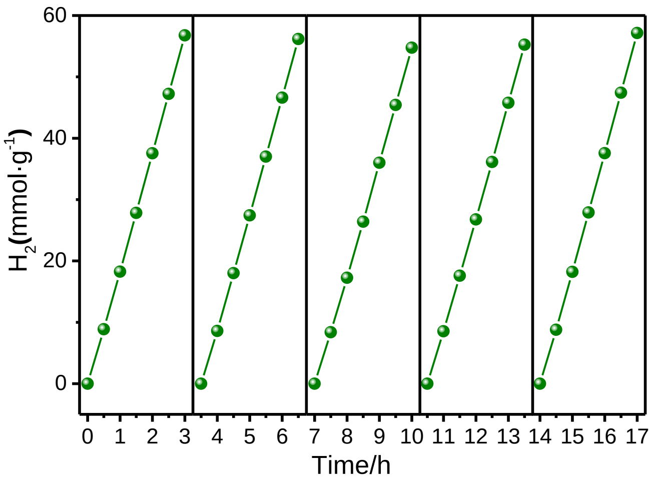

Supplementary Fig. 21 Stability study of TiO_2-10 for photocatalytic H_2 generation.

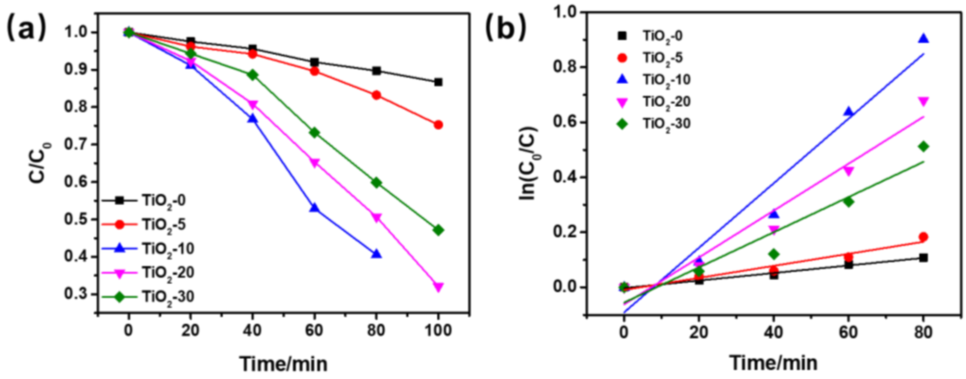

Supplementary Fig. 22 Photodegradation of phenol using TiO_2-*m*. (a) Photodegradation *vs.* reaction time; (b) ln(C_0/C) *vs.* irradiation time (C is the corresponding degradative concentration and C_0 is initial concentration of phenol).

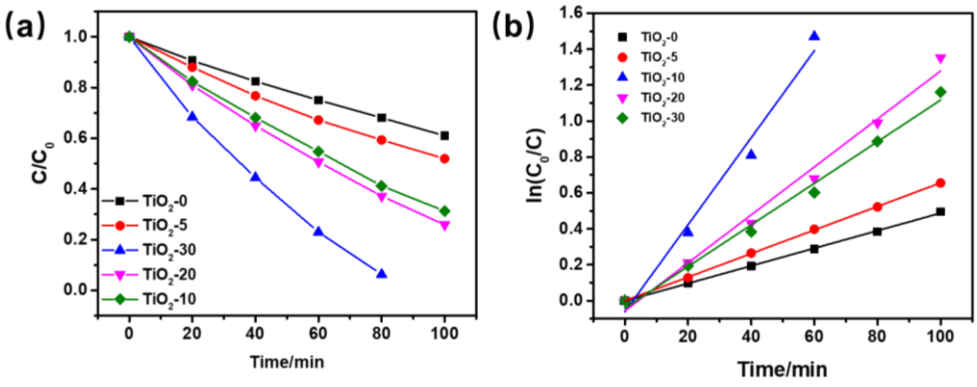

Supplementary Fig. 23 Photodegradation of MO using TiO_2-*m*. (a) Photodegradation *vs.* reaction time; (b) ln(C_0/C) *vs.* irradiation time (C is the corresponding degradative concentration and C_0 is initial concentration of MO).

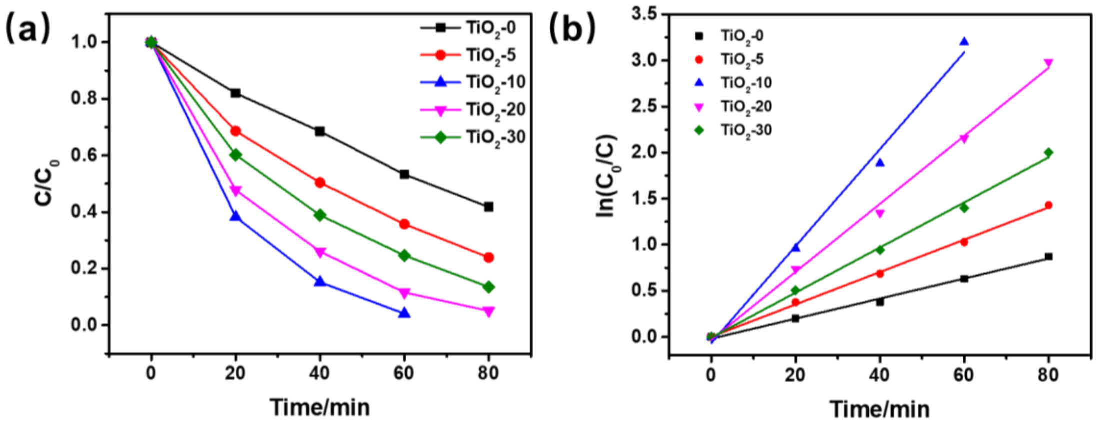

Supplementary Fig. 24 Photodegradation of RhB using TiO_2-*m*. (a) Photodegradation *vs.* reaction time; (b) ln(C_0/C) *vs.* irradiation time (C is the corresponding degradative concentration and C_0 is initial concentration of RhB).

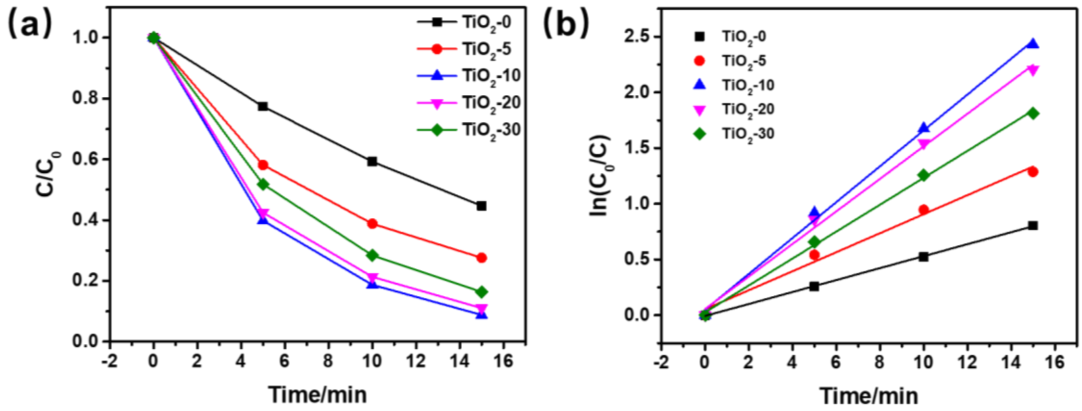

Supplementary Fig. 25 Photodegradation of Rose Bengal using TiO_2-*m*. (a) Photodegradation *vs.* reaction time; (b) ln(C_0/C) *vs.* irradiation time (C is the corresponding degradative concentration and C_0 is initial concentration of Rose Bengal).

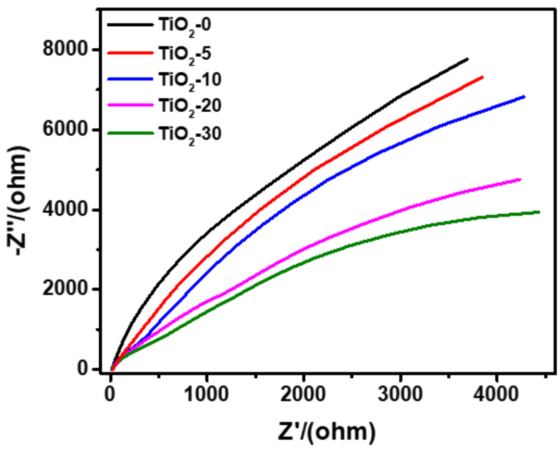

Supplementary Fig. 26 EIS Nyquist plots of TiO_2-*m* under light irradiation.

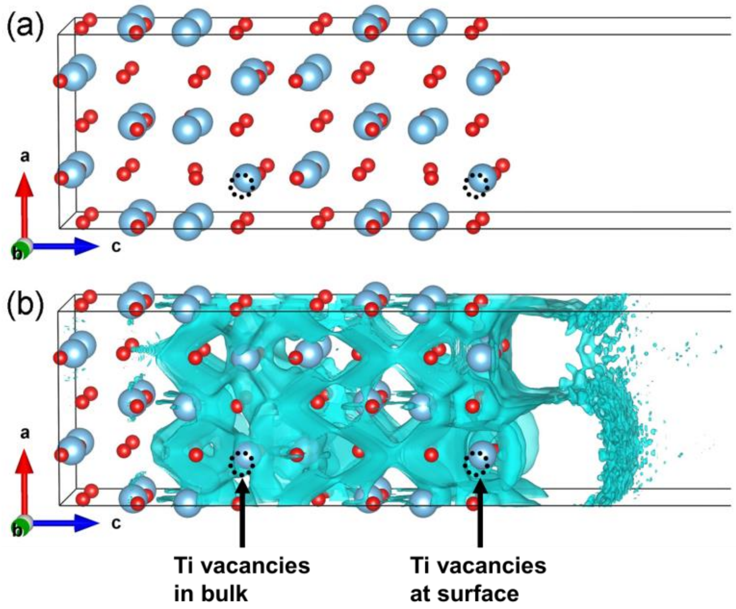

Supplementary Fig. 27 The calculation of surface and bulk spatial spin polarization (SSP). (a) The 3D geometry and (b) 3D spatial spin polarization in TiO_2 at V_{Ti}=6.25%. The energy interval is [E_F-0.2 eV, E_F]. The specific iso-surface SSP value is -95%. The dashed black circles indicate the positions of Ti vacancies.

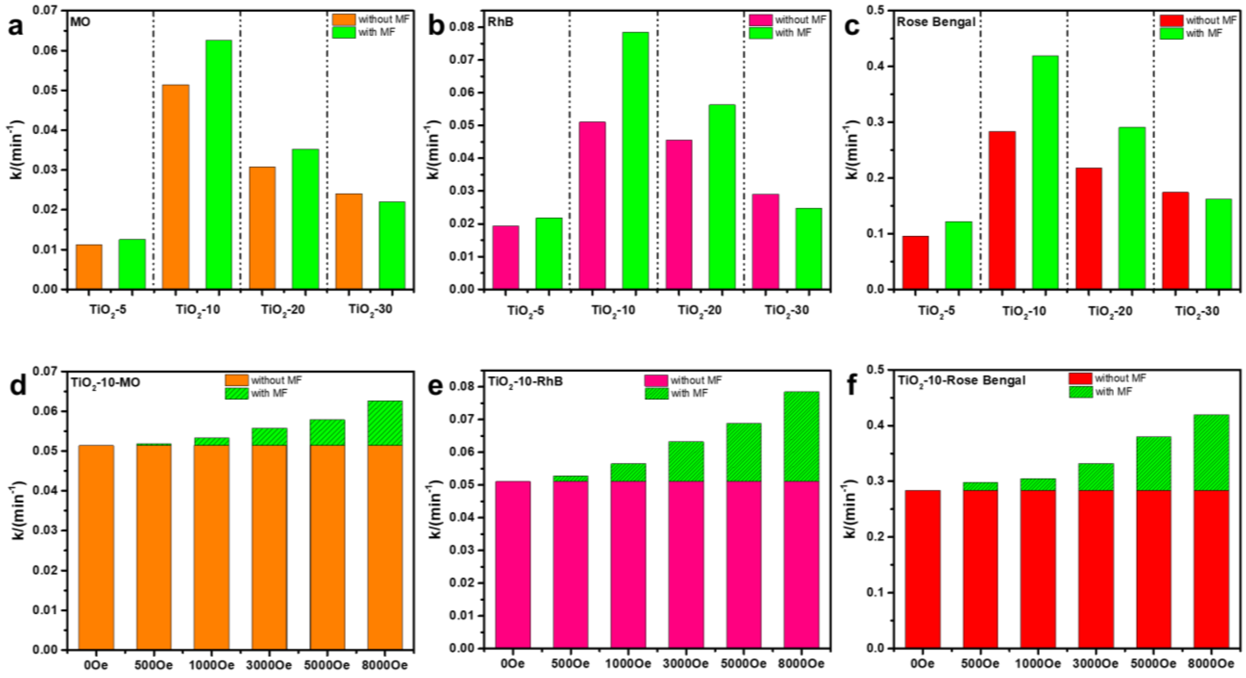

Supplementary Fig. 28 The effect of magnetic field on photodegradation. Photodegradation rates of (a) MO, (b) RhB, (c) Rose Bengal without and with magnetic field (8000Oe) of TiO_2-*m*. Photodegradation rates of (d) MO, (e) RhB, (f) Rose Bengal under different magnetic field intensity of TiO_2-10.

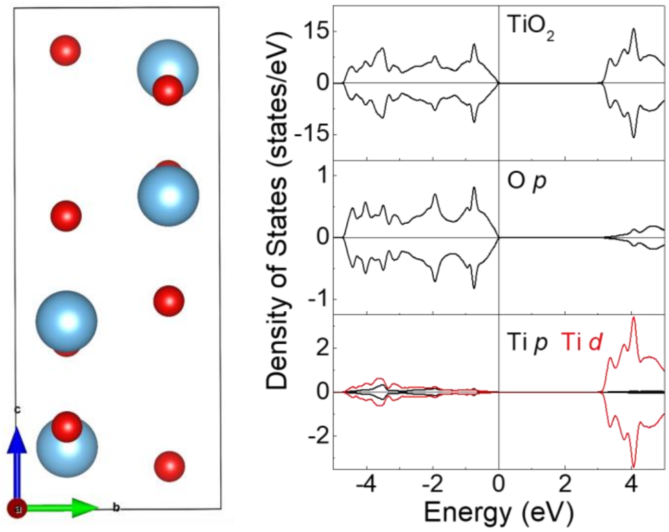

Supplementary Fig. 29 Calculated supercell and density of states of stoichiometric TiO_2.

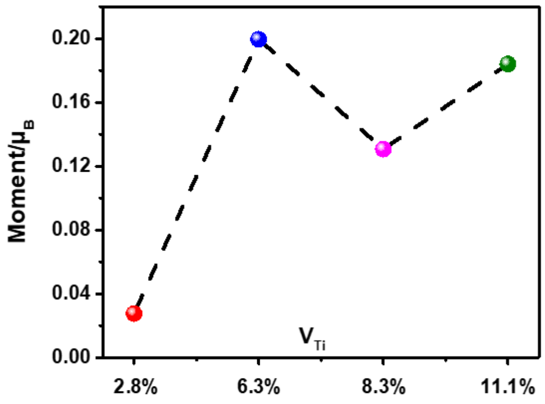

Supplementary Fig. 30 Calculated moment of defected TiO_2.

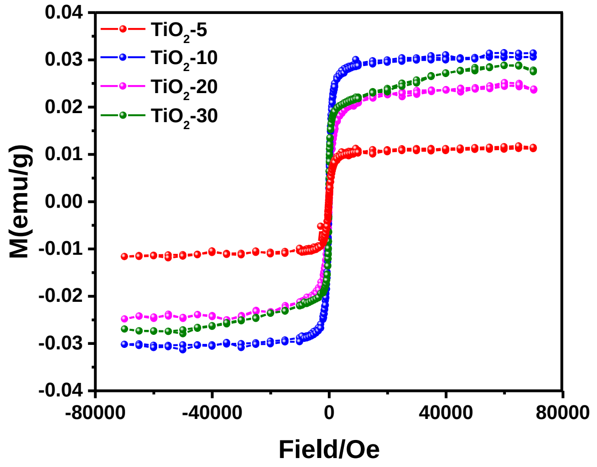

Supplementary Fig. 31 Magnetization (M-H) curves measured at 300 K of TiO_2-*m*.

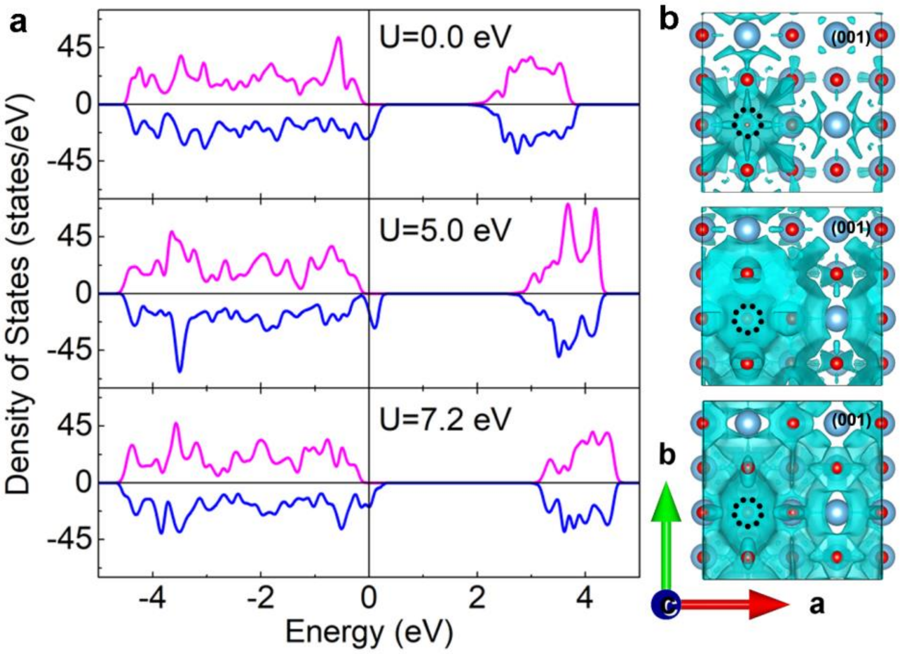

Supplementary Fig. 32 The effect of Hubbard *U* on the spatial spin polarization (SSP). The DOS (a) and (001)-planar SSP (b) of TiO_2 at V_{Ti}=6.25% under the Hubbard $U_{\mathrm{Ti},\,3d}$ of 0.0 eV, 5.0 eV and 7.2 eV, respectively. The Fermi level is indicated by the vertical lines and set to zero. The energy interval is [E_F-0.2 eV, E_F]. The specific iso-surface SSP value is -95%. The dashed black circles indicate the positions of Ti vacancies in TiO_2.

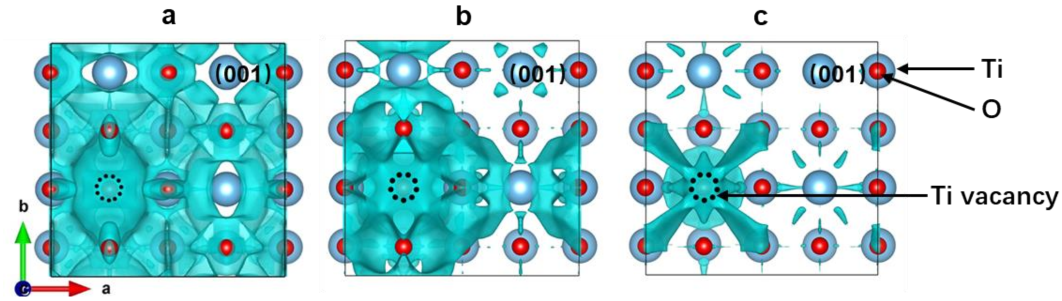

Supplementary Fig. 33 The effect of energy interval on the spatial spin polarization (SSP). (001)-planar SSP in TiO_2 at V_{Ti}=6.25% in the energy interval of (a) [E_F-0.2 eV, E_F], (b) [E_F-0.3 eV, E_F] and (c) [E_F-0.4 eV, E_F], respectively. The specific iso-surface SSP value is -95%.

As shown in **Supplementary Fig. 33**, the energy intervals don't affect the negative SSP results, but affects the spatial distributions of spin polarization. The electrons participating the photocatalysis is around the Fermi level, so we choose the energy range of [E_F-0.2 eV, E_F] for DFT calculation.

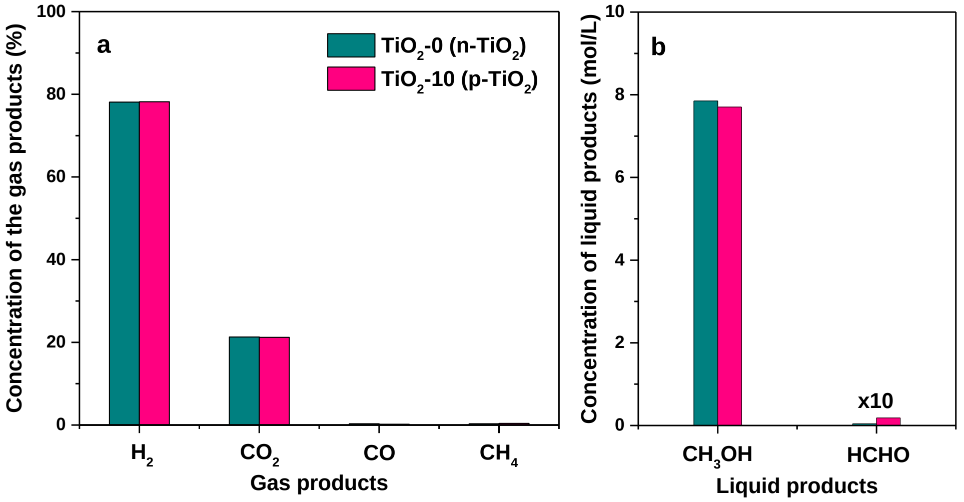

Supplementary Fig. 34 Products analysis of photocatalysis. The gas products (a) and liquid products (b) of photocatalytic hydrogen generation and methanol oxidation, detected by gas chromatography and liquid chromatography, respectively. The reaction conditions: 100 mL aqueous solution containing methanol (30 $_{vol}$%); 10 mg catalysts (TiO_2-0 or TiO_2-10); 0.5 °C ± 0.1 °C; reaction time: 8 h. It is worth noting that no formic acid and other byproducts are detected.

Supplementary Tables

Supplementary Table 1 Lattice constants and Ti/O molar ratio of TiO_2-*m*.

Sample	*a*(Å)	*b*(Å)	*c*(Å)	Ti/O ratio		Defect concentration	
				Bulk	Surface	bulk	surface
21-1272[a](TiO_2)	3.785[a]	3.785[a]	9.514[a]	0.500	0.500	0%	0%
TiO_2-0	3.785[b]	3.785[b]	9.509[b]	0.496[c]	0.499[d]	0.8%	0.2%
TiO_2-5	3.786[b]	3.786[b]	9.505[b]	0.486[c]	0.485[d]	2.8%	3.0%
TiO_2-10	3.787[b]	3.787[b]	9.503[b]	0.468[c]	0.462[d]	6.4%	7.6%
TiO_2-20	3.786[b]	3.786[b]	9.501[b]	0.453[c]	0.447[d]	9.4%	10.6%
TiO_2-30	3.787[b]	3.787[b]	9.500[b]	0.447[c]	0.445[d]	10.6%	11.0%

[a]Standard JCPDS card of anatase TiO_2. [b]Fitted from Rietveld refinement of XRD patterns. [c]Calculated from chemical titration. [d]Calculated from XPS measurement.

Supplementary Table 2 Parameters of N_2 adsorption-desorption isotherms for TiO_2-*m*.

Sample	Surface area (m^2/g)	Pore size (nm)	Pore volume (cm^3/g)
TiO_2-0	64.66	12.14	0.2573
TiO_2-5	81.42	10.78	0.2860
TiO_2-10	104.22	13.02	0.4392
TiO_2-20	85.49	14.71	0.4513
TiO_2-30	91.71	16.93	0.5560

Supplementary Table 3 Positron lifetimes and relative intensities of TiO_2-*m*.

Sample	τ_1(ps)	I_1(%)	τ_2(ps)	I_2(%)	τ_3(ps)	I_3(%)
TiO_2-0	264.0	21.5	352	78.5	6100	NA
TiO_2-5	315.6	80.0	339.4	14.8	430.7	5.2
TiO_2-10	319.0	96.0	566	3.8	2820	0.2
TiO_2-20	321.5	90.3	374.8	9.4	554.1	0.3
TiO_2-30	314.8	87.5	446.6	11.7	2427	0.8

Supplementary Table 4 Fitted parameters of Ti K-edge EXAFS curves for TiO_2-*m*.

Sample	path	R(Å)[a]	S_0^2 [b]	N[c]	S^2(Å^{-2})[d]	R_{eff}(Å)[e]
	Ti-O_1	1.94487		4	0.00538	1.93230
TiO_2-0	Ti-O_2	1.99137	0.8	2	0.00538	1.97880
	Ti-Ti_1	2.99700		4	0.00611	3.03640
	Ti-O_1	1.94489		4	0.00544	1.93230
TiO_2-5	Ti-O_2	1.99139	0.8	2	0.00544	1.97880
	Ti-Ti_1	2.99169		3.87	0.00640	3.03640
	Ti-O_1	1.94675		4	0.00577	1.93230
TiO_2-10	Ti-O_2	1.99325	0.8	2	0.00577	1.97880
	Ti-Ti_1	2.99572		3.82	0.00690	3.03640
	Ti-O_1	1.94948		4	0.00590	1.93230
TiO_2-20	Ti-O_2	1.99598	0.8	2	0.00590	1.97880
	Ti-Ti_1	2.99545		3.79	0.00660	3.03640
	Ti-O_1	1.95025		4	0.00599	1.93230
TiO_2-30	Ti-O_2	1.99676	0.8	2	0.00599	1.97880
	Ti-Ti_1	2.99506		3.75	0.00650	3.03640

[a]Actual distance to neighboring atom. [b]Amplitude attenuation factor. [c]Coordination number of neighboring atom. [d]Mean-square disorder of neighbor distance. [e]Theoretical distance to neighboring atom.

Supplementary Table 5 Parameters of Mott-Schottky plots for TiO_2-*m*.

Sample	Carrier type	Flat band potential (V vs. Ag/AgCl)	Carrier density (cm^{-3})
TiO_2-0	n	-0.42	8.79×10^{11}
TiO_2-5	p	1.41	1.37×10^{13}
TiO_2-10	p	1.45	2.51×10^{13}
TiO_2-20	p	1.49	5.16×10^{13}
TiO_2-30	p	1.55	1.23×10^{14}

The density of carriers is calculated by means of Mott-Schottky formula

$$N_d = \frac{2}{\varepsilon\varepsilon_0 e}\frac{d(E-E_{fb}-\frac{kT}{e})}{d(\frac{1}{C^2})} \qquad (2)$$

where N_d is charge carrier densities, ε_0 is vacuum permittivity (8.854×10^{-14} F/cm), ε is permittivity of anatase TiO_2, e equals to 1.6×10^{-19} C and $d(E-E_{fb}-kT/e)/d(1/C^2)$ is the reciprocal of the slope of the Mott–Schottky curves.

Supplementary Table 6 Average fluorescent lifetime of TiO_2-m.

Sample	A_1(%)	τ_1(ns)	A_2(%)	τ_2(ns)	τ_a(ns)
TiO_2-0	21.63	0.323	78.37	4.10	4.02
TiO_2-5	20.19	0.476	79.81	4.33	4.23
TiO_2-10	10.38	0.332	89.62	4.78	4.74
TiO_2-20	16.16	0.336	83.84	4.62	4.56
TiO_2-30	17.11	0.335	82.98	4.55	4.48

All the PL decays are fitted [4]:

$$\mathrm{I(t)} = A_1 e^{-t/\tau_1} + A_2 e^{-t/\tau_2} \quad (3)$$

where τ_1 and τ_2 are the fluorescent lifetime, and A_1 and A_2 are the corresponding amplitude. Supplementary Table 6 summarizes the average fluorescent lifetime, given by

$$\tau_a = \frac{A_1\tau_1^2 + A_2\tau_2^2}{A_1\tau_1 + A_2\tau_2} \quad (4)$$

Supplementary Table 7 The supercell size and atomic numbers of normal and Ti-defected TiO_2.

V_{Ti}[a]	a(Å)	b(Å)	c(Å)	atom
0.0%	3.916	3.916	9.606	Ti_4O_8
6.25%	7.865	7.865	9.497	$Ti_{15}O_{32}$

[a] V_{Ti} indicates the content of Ti vacancies in TiO_2.

Supplementary Methods

Quantum efficiency test and calculation. The apparent quantum efficiency at 380 nm was measured using the same experimental setup with that for photocatalysis. 10 mg catalyst were dispersed in 100 mL aqueous solution containing methanol (30 $_{vol}$%). 0.5 $_{wt}$% Pt was introduced as cocatalyst by *in-situ* photodeposition. The temperature of reaction solution was maintained at 0.5 °C ± 0.1 °C. The photocatalyst aqueous solution was irradiated by a 300 W Xe lamp with a 380 nm band-pass optical filter with full width at half maximum of 15 nm. The average intensity of irradiation was determined to be 2.0 mW/cm^2 by a UV-A radiometer (Photoelectric Instrument Factory, Beijing Normal University) and the irradiation area was 20 cm^2. The amount of H_2 molecules generated in 2 h was used for quantum efficiency evaluation on the basis of the following formula:

$$\mathrm{AQE} = \frac{2\times \text{the number of evolved } \mathrm{H_2} \text{ molecules}}{\text{the number of incident photons}} \times 100\% \quad (5)$$

where AQE is the apparent quantum efficiency.

Gaseous products and liquid products analysis of the photocatalytic reaction. The reaction was carried out under ultraviolet light in a reaction device similar to that for photocatalysis but with two sampling ports (gas phase and liquid phase). 10 mg catalyst were dispersed in 100 mL aqueous solution containing methanol (30 $_{vol}$%). 1 $_{wt}$% Pt was introduced as cocatalyst by *in-situ* photodeposition. The temperature of reaction solution was maintained at 0.5 °C ± 0.1 °C. The analysis of the gaseous reaction mixtures at 8 h was carried out using a gas chromatograph (Shimadzu GC-2010Plus) equipped with a barrier discharge ionization detector (BID) and column (C47839-06). Liquid products were quantitatively analyzed by a high performance liquid chromatography (HPLC) (Agilent 1260 Infinity LC) with a DAD detector and C18 column (ZORBAX Eclipse XDB-C18).

Supplementary References

1. Hu, C., et al. Edge sites with unsaturated coordination on core-shell $Mn_3O_4@Mn_xCo_{3-x}O_4$ nanostructures for electrocatalytic water oxidation. *Adv. Mater.* **29**, 1701820 (2017).
2. Li, X., et al. Structures and magnetic properties of p-type Mn:TiO_2 dilute magnetic semiconductor thin films. *J. Appl. Phys.* **106**, 043913 (2009).
3. Zhang, H., et al. Novel TiO_2/PEGDA hybrid hydrogel prepared *in-situ* on tumor cells for effective photodynamic therapy. *ACS Appl. Mater. Interfaces* **5**, 12317-12322 (2013).
4. Shepherd, D.P., Sambur, J.B., Liang, Y.-Q., Parkinson, B.A. & Van Orden, A. *In-situ* studies of photoluminescence quenching and photocurrent yield in quantum dot sensitized single crystal TiO_2 and ZnO electrodes. *J. Phys. Chem. C* **116**, 21069-21076 (2012).
